# The role of NMR-based circulating metabolic biomarkers in development and risk prediction of new onset type 2 diabetes

**DOI:** 10.1038/s41598-022-19159-8

**Published:** 2022-09-05

**Authors:** Fiona Bragg, Christiana Kartsonaki, Yu Guo, Michael Holmes, Huaidong Du, Canqing Yu, Pei Pei, Ling Yang, Donghui Jin, Yiping Chen, Dan Schmidt, Daniel Avery, Jun Lv, Junshi Chen, Robert Clarke, Michael R. Hill, Liming Li, Iona Y. Millwood, Zhengming Chen

**Affiliations:** 1 Clinical Trial Service Unit and Epidemiological Studies Unit (CTSU), Nuffield Department of Population Health, University of Oxford, BDI Building, Old Road Campus, Oxford, OX3 7LF UK; 2 Medical Research Council Population Health Research Unit, Nuffield Department of Population Health, University of Oxford, Oxford, UK; 3 Fuwai Hospital Chinese Academy of Medical Sciences, National Center for Cardiovascular Diseases, Beijing, China; 4 Department of Epidemiology and Biostatistics, School of Public Health, Peking University Health Science Center, Beijing, China; 5 Peking University Center for Public Health and Epidemic Preparedness & Response, Beijing, China; 6 Chinese Academy of Medical Sciences, Beijing, 102308 China; 7 Hunan Centre for Disease Control and Prevention, Furong Mid Road, Changsha, Hunan China; 8 China National Center for Food Safety Risk Assessment, Beijing, China

**Keywords:** Biomarkers, Endocrine system and metabolic diseases, Risk factors, Epidemiology

## Abstract

Associations of circulating metabolic biomarkers with type 2 diabetes (T2D) and their added value for risk prediction are uncertain among Chinese adults. A case-cohort study included 882 T2D cases diagnosed during 8-years’ follow-up and a subcohort of 789 participants. NMR-metabolomic profiling quantified 225 plasma biomarkers in stored samples taken at recruitment into the study. Cox regression yielded adjusted hazard ratios (HRs) for T2D associated with individual biomarkers, with a set of biomarkers incorporated into an established T2D risk prediction model to assess improvement in discriminatory ability. Mean baseline BMI (SD) was higher in T2D cases than in the subcohort (25.7 [3.6] vs. 23.9 [3.6] kg/m^2^). Overall, 163 biomarkers were significantly and independently associated with T2D at false discovery rate (FDR) controlled *p* < 0.05, and 138 at FDR-controlled *p* < 0.01. Branched chain amino acids (BCAA), apolipoprotein B/apolipoprotein A1, triglycerides in VLDL and medium and small HDL particles, and VLDL particle size were strongly positively associated with T2D (HRs 1.74–2.36 per 1 SD, p < 0.001). HDL particle size, cholesterol concentration in larger HDL particles and docosahexaenoic acid levels were strongly inversely associated with T2D (HRs 0.43–0.48, *p* < 0.001). With additional adjustment for plasma glucose, most associations (n = 147 and n = 129 at *p* < 0.05 and *p* < 0.01, respectively) remained significant. HRs appeared more extreme among more centrally adipose participants for apolipoprotein B/apolipoprotein A1, BCAA, HDL particle size and docosahexaenoic acid (p for heterogeneity ≤ 0.05). Addition of 31 selected biomarkers to an established T2D risk prediction model modestly, but significantly, improved risk discrimination (c-statistic 0.86 to 0.91, p < 0.001). In relatively lean Chinese adults, diverse metabolic biomarkers are associated with future risk of T2D and can help improve established risk prediction models.

## Introduction

Worldwide, over 460 million adults are estimated to be living with diabetes (mostly type 2 diabetes [T2D]), and over one quarter of these live in China^1^, where diabetes affects > 10% of the adult population^2^. Effective prevention of T2D is reliant on accurate prediction of disease risk and understanding of underlying aetiological mechanisms. T2D is characterised by disturbances across multiple metabolic pathways, yet existing risk prediction models typically rely on a limited number of relatively distal variables within these pathways (e.g., glycaemia, blood pressure, lipidaemia)^3^. The human metabolome (representing the downstream end-products of genetic, epigenetic and environmental pathways) serves as an efficient tool for simultaneously quantifying metabolites across multiple pathways. Furthermore, its characterisation in diverse populations has the potential to identify more proximal risk markers, permitting earlier detection of T2D risk and precision prevention.

Many prospective studies have reported significant associations of circulating metabolic biomarkers with T2D risk, including branched chain and aromatic amino acids, hexoses, lipids, and phospholipids^4–15^. However, they were constrained by relatively small sample sizes, investigation of limited numbers of biomarkers, and use of less standardised metabolic profiling techniques, which may account for inconsistent findings between studies^5–7,11–13,16–18^. Moreover, much existing evidence is based on Western studies, with limited data available from other populations, including China, where diabetes prevalence is high despite the relatively lean population, where diabetes onset is typically at a younger age and lower body mass index (BMI) than in more widely-studied Western populations, and where there is marked heterogeneity in diabetes prevalence (e.g., between urban and rural locations)^19^. Appropriate understanding of how metabolic biomarkers associate with T2D risk across diverse populations, including populations with different levels and distributions of adiposity, may advance understanding of T2D aetiology and improve our ability to accurately predict T2D risk.

To address existing evidence gaps, we investigated the prospective associations of > 200 circulating metabolic biomarkers, measured using a replicable, targeted, high-throughput NMR-metabolomics platform, with risk of incident T2D during 8 years’ follow-up in a case-cohort study within the China Kadoorie Biobank (CKB), and assessed whether factors such as age, sex and adiposity modify these associations. We further examined the discriminatory ability of these biomarkers to improve T2D risk discrimination.

## Results

Baseline characteristics of T2D cases (n = 882, including those in the subcohort) and subcohort participants (n = 789) are presented in Table 1. Cases had a higher mean (SD) age at baseline (55.1 [9.6] vs. 51.9 (10.6) years), and a lower proportion lived in urban areas (38.8% vs. 49.0%), but the proportion of women was similar among T2D cases and subcohort participants (63.0% vs. 61.9%, respectively). Participants with T2D were, on average, less highly educated, more likely to have a family history of diabetes, and less likely to regularly consume fresh fruit or dairy products. They had higher mean BMI (25.7 [3.6] vs. 23.9 [3.6] kg/m^2^) and waist circumference (WC) (85 [10] vs. 80 [10] cm) than subcohort participants.

**Table 1:** Baseline characteristics of T2D cases and subcohort participants.

	T2D cases (n = 882)	Subcohort (n = 789)
**Age, sex and socioeconomic factors**		
Mean age (SD), years	55.1 (9.6)	51.9 (10.6)
Women, %	63.0	61.9
≥ 6 years of education, %	30.3	54.8
Living in urban area, %	38.8	49.0
**Lifestyle factors**		
Ever-regular smoker, %	30.0	29.0
Ever-regular alcohol drinker, %	18.9	15.7
Mean physical activity (SD), MET-h/d	21 (14)	23 (15)
Regular consumption^a^, %		
Fresh fruit	23.7	30.7
Red meat	47.6	49.3
Fish	9.3	10.0
Dairy products	7.7	13.4
**Anthropometry, blood pressure and heart rate, mean (SD)**		
BMI, kg/m^2^	25.7 (3.6)	23.9 (3.6)
WC, cm	85 (10)	80 (10)
HC, cm	93 (7)	91 (7)
WHR	0.92 (0.07)	0.88 (0.07)
Body fat percentage	32 (9)	29 (8)
SBP, mmHg	142 (23)	131 (22)
Resting heart rate, bpm	80 (12)	79 (11)
**Self-reported statin use, %**	0.6	0.3
**Self-reported poor health, %**	10.2	9.4
**Family history of diabetes**^**b**^**, %**	10.1	7.7
**Mean fasting time (SD), hours**	6.2 (5.4)	5.2 (5.1)

*BMI* body mass index, *HC* hip circumference, *MET-h/d* metabolic equivalent of task hours per day, *SBP* systolic blood pressure, *T2D* type 2 diabetes, *WC* waist circumference, *WHR* waist-to-hip ratio.

^a^Consumption on at least 4–6 days per week.

^b^History of diabetes among first-degree relatives.

Correlations between directly measured metabolic biomarkers are presented in Supplementary Fig. S1. Overall, 178 of the 225 metabolic biomarkers, across multiple molecular pathways, were associated with risk of incident T2D at false discovery rate (FDR) controlled *p* < 0.05 after adjustment for age, sex, study area, education and fasting time, of which 134 were significant at FDR-controlled *p* < 0.01 (Supplementary Table S1, Supplementary Figs. S2, S3). After additional adjustment for lifestyle factors, family history of diabetes and adiposity, 163 biomarkers remained statistically significantly associated with T2D at *p* < 0.05 and 138 at *p* < 0.01. Further adjustment for random plasma glucose (RPG) moderately attenuated most associations, but the majority (n = 147 at *p* < 0.05; n = 129 at *p* < 0.01) remained statistically significant.

### Lipoproteins and incident T2D

**Figure 1:**
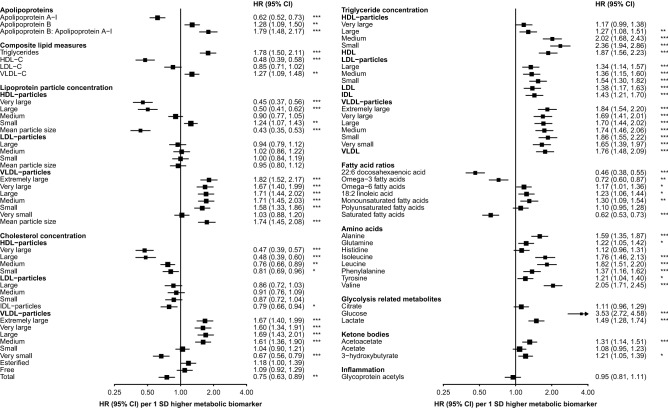
Associations of circulating metabolic biomarkers with risk of incident type 2 diabetes. Adjusted for age, sex, study area, education, fasting time, smoking, alcohol, physical activity, dietary factors, family history of diabetes, BMI and waist circumference. Squares represent the HR per 1 SD higher metabolic biomarker. Horizontal lines represent the corresponding 95% CI. Fatty acid ratios represent ratios of individual to total fatty acids. *p ≤ 0.05, **p ≤ 0.01, ***p ≤ 0.001 after adjustment for multiple testing using Benjamini–Hochberg correction.

There were positive associations with incident T2D risk of apolipoprotein B/apolipoprotein A1 (1.79 [95% CI 1.48–2.17] per 1 SD higher), triglyceride (1.78 [1.50–2.11]) and VLDL-cholesterol (1.27 [1.09–1.48]) concentrations, as well as VLDL particle size (1.74 [1.45–2.08]) (Fig. 1). The concentration of HDL-cholesterol (0.48 [0.39–0.58]) and HDL particle size (0.43 [0.35–0.53]) showed inverse associations with risk of T2D.

Higher triglyceride concentrations in all lipoprotein subclasses were associated with higher T2D risks. These risks were moderately stronger for triglyceride concentrations in small (2.36 [95% CI 1.94–2.86]) and medium (2.02 [1.68–2.43]) HDL particles. Triglyceride concentrations in VLDL particles were associated with 65–86% higher risk per 1 SD, with a similar strength of association irrespective of particle size. Each 1 SD increment in cholesterol concentration in medium to extremely large VLDL particles was associated with 60–69% higher risk of T2D, while cholesterol in very small VLDL particles was associated with ~ 30% lower risk. The inverse associations of cholesterol concentrations in large (0.48 [0.39–0.60]) and very large (0.47 [0.39–0.57]) HDL particles were stronger than those of cholesterol concentrations in smaller HDL particles.

### Amino acids and incident T2D

Branched chain amino acid (BCAA) (leucine, isoleucine, valine) concentrations were strongly positively associated with risk of incident T2D, ranging from an adjusted HR of 1.76 (95% CI 1.46–2.13) for isoleucine to 2.05 (1.71–2.45) for valine (Fig. 1). Moderately weaker associations (20–60% higher risk per 1 SD increment) were observed with other measured amino acids, with the exception of histidine which showed no clear association with T2D risk.

### Fatty acids and incident T2D

Total fatty acid concentration was positively associated with risk of T2D (1.45 [95% CI 1.23–1.71] per 1 SD) (Supplementary Table S1). Higher absolute concentrations of linoleic acid (1.72 [1.43–2.07]), as well as omega-6 (1.71 [1.43–2.05]), monounsaturated (1.47 [1.25–1.73]), polyunsaturated (1.61 [1.35–1.93]) and saturated (1.26 [1.09–1.47]) fatty acids were associated with higher T2D risks. There was no association of overall omega-3 fatty acids with T2D risk (1.05 [0.88–1.25]), but there was an inverse association of docosahexanoic acid (0.66 [0.55–0.79]). When relative fatty acid concentrations (i.e., relative to total fatty acid concentration) were examined, the associations of linoleic acid (1.23 [1.06–1.44]) and omega-6 (1.17 [1.01–1.36]) and monounsaturated (1.30 [1.09–1.54]) fatty acids persisted, but were attenuated (Fig. 1). There were inverse associations of relative concentrations of saturated (0.62 [0.53–0.73]) and omega-3 (0.72 [0.60–0.87]) fatty acids, and of docosahexaenoic acid (0.46 [0.38–0.55]). There was no clear association of polyunsaturated fatty acids (1.10 [0.95–1.28]).

### Ketone bodies, glycolysis and inflammation and incident T2D

Glucose levels were strongly positively associated with future T2D risk (3.53 [95% CI 2.72–4.58] per 1 SD higher) (Fig. 1). There were weaker positive associations of lactate (1.49 [1.28–1.74]) and of quantified ketone bodies (acetoacetate: 1.31 [1.14–1.51]; 3-hydroxybutyrate: 1.21 [1.05–1.39]). There was no clear association, overall, between glycoprotein acetyl concentration and risk of incident T2D.

### Influence of obesity on metabolic biomarker associations with incident T2D

**Figure 2:**
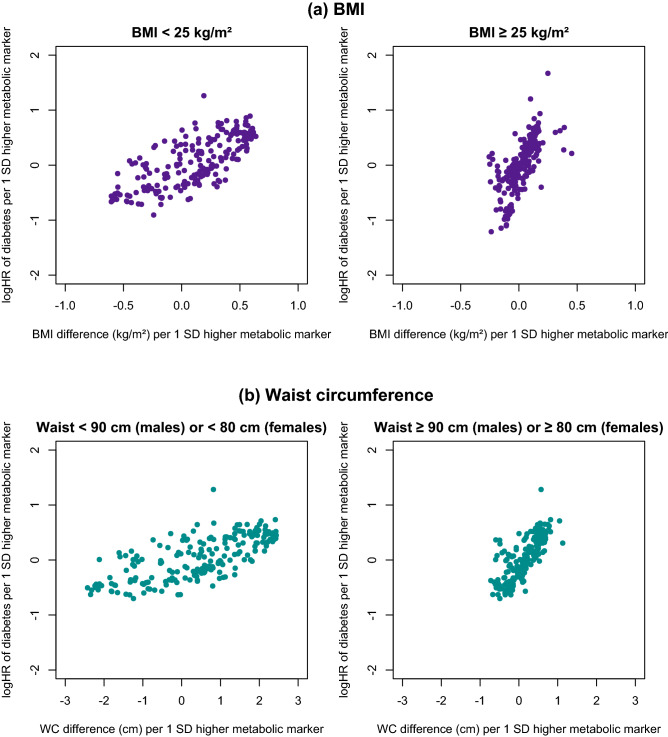
Comparison of differences in adiposity associated with 1-SD higher metabolic biomarkers vs. log-hazard ratios (HRs) for incident type 2 diabetes associated with 1-SD higher metabolic biomarkers, stratified by (**a**) BMI and (**b**) waist circumference. Estimates on the x-axis are the coefficients of linear regression of metabolic biomarkers on (**a**) BMI and (**b**) waist circumference. Estimates on the y-axis are the coefficients of Cox regression of type 2 diabetes on metabolic biomarkers. All estimates are adjusted for age, sex, study area, education, fasting time, smoking, alcohol, physical activity, dietary factors and family history of diabetes. *BMI* body mass index, *WC* waist circumference.

Metabolic biomarkers displaying stronger associations with adiposity measures also tended to be more strongly associated with risk of incident T2D (Fig. 2). Moreover, among individuals with central obesity (WC ≥ 90 cm in men and ≥ 80 cm in women), when compared with those without central obesity, each 1 SD increment in metabolic biomarkers tended to be associated with smaller differences in WC, but similar or greater differences in risk of T2D. A similar, albeit less extreme, pattern was observed for BMI.

The HRs were more extreme among participants with, than without, central obesity for apolipoprotein B/apolipoprotein A1 (HR 2.99 vs. 1.45; *p* ≤ 0.05) (Supplementary Fig. S4) and BCAA (leucine 2.46 vs. 1.73, *p* ≤ 0.01; isoleucine 2.49 vs. 1.67, *p* ≤ 0.05; valine 2.30 vs. 2.04, *p* ≤ 0.05) (Supplementary Fig. S5) with T2D. Similar findings were also evident for certain biomarkers showing inverse associations with T2D, including HDL particle size (0.27 vs. 0.58, *p* ≤ 0.001) and docosahexaenoic acid (0.46 vs. 0.53, *p* ≤ 0.05). Associations of other lipid measures and of larger VLDL particles were also modestly, but non-significantly, more extreme, as were associations across the BMI strata examined.

Lipids, apolipoproteins and lipoprotein particle concentrations tended to be more strongly associated with T2D among younger participants (Supplementary Figs. S6, S7). However, the associations of other metabolic biomarkers differed little by age, and there were no clear sex (Supplementary Figs. S8, S9) or urban–rural (Supplementary Figs. S10, S11) differences. The associations remained largely unchanged in sensitivity analyses excluding the first 2 years of follow-up (Supplementary Fig. S12).

### T2D risk prediction

Addition of 31 selected circulating metabolic biomarkers (including amino acids, fatty acids, lipoproteins, and inflammatory and glycolysis-related biomarkers) (Table 2) to an established T2D risk score^20^ significantly improved risk discrimination, increasing the c-statistic from 0.86 (95% CI 0.84–0.88) to 0.91 (0.90–0.93) (*p* for difference < 0.001). The performance of this model was comparable across population subgroups defined by age, sex and adiposity.

**Table 2:** Discriminatory ability of prediction models for incident type 2 diabetes.

Model		C-statistic (95% CI)
Base model^a^		0.86 (0.84–0.88)
**Enhanced model: base model plus selected metabolic biomarkers**^**b**^		
Total population		0.91 (0.90–0.93)
Population subgroups		
BMI	< 25.0 kg/m^2^	0.91 (0.89–0.93)
	≥ 25.0 kg/m^2^	0.91 (0.89–0.93)
WC	< 90 cm in men and < 80 cm in women	0.91 (0.88–0.93)
	≥ 90 cm in men and ≥ 80 cm in women	0.92 (0.90–0.94)
Sex	Men	0.91 (0.89–0.94)
	Women	0.92 (0.91–0.94)
Age	< 55 years	0.93 (0.91–0.94)
	≥ 55 years	0.90 (0.88–0.93)

*BMI* body mass index, *WC* waist circumference.

^a^Age, sex, study area, fasting time, body mass index, family history of diabetes, education, blood pressure, resting heart rate, plasma glucose, triglycerides, statin use.

^b^Concentrations of linoleic acid, docosahexaenoic acid, 3-hydroxybutyrate, apolipoprotein A-I, esterified cholesterol, free cholesterol in very small VLDL, glycoprotein acetyls, lactate, phosphatidylcholine, phospholipids in IDL, total cholesterol in very large HDL, total lipids in large HDL, total lipids in small VLDL, triglycerides in large HDL, triglycerides in small HDL, valine, ratios of docosahexaenoic acid to total fatty acids, apolipoprotein B to apolipoprotein A-I, cholesterol esters to total lipids in medium HDL, cholesterol esters to total lipids in small VLDL, free cholesterol to total lipids in large HDL, free cholesterol to total lipids in medium HDL, free cholesterol to total lipids in small HDL, free cholesterol to total lipids in very small VLDL, phospholipids to total lipids in large LDL, phospholipids to total lipids in large VLDL, phospholipids to total lipids in small HDL, triglycerides to total lipids in IDL, triglycerides to total lipids in small VLDL, mean HDL particle diameter.

## Discussion

This prospective, population-based study represents the most comprehensive assessment of the metabolomic profile of future T2D risk in the Chinese population. There were strong positive associations of BCAA, apolipoprotein B/apolipoprotein A1, triglycerides, and VLDL particle size, and inverse associations of omega-3 fatty acids, HDL particle size and cholesterol concentrations in large HDL particles. The associations of several of the biomarkers most strongly related to future T2D risk were more extreme among participants with central obesity. When combined with traditional risk predictors, including glycaemia, circulating metabolic biomarkers significantly improved prediction of T2D over an average 8-year period.

The associations of BCAAs with incident T2D were among the strongest observed, and were qualitatively, and broadly quantitatively, consistent with previous study findings^4,5,12,13^. For example, a meta-analysis with ~ 1500 cases of incident T2D from seven individual prospective, predominantly Western population, studies, found adjusted RRs for T2D of 1.36, 1.36 and 1.35 per 1 SD higher isoleucine, leucine and valine, respectively^4^. Similarly, in a nested case–control study in China, comprising ~ 1500 incident T2D cases and a similar number of controls, there were positive associations of leucine/isoleucine and valine concentrations with T2D, with adjusted RRs comparing top vs. bottom quartiles of 1.75 and 1.54, respectively^12^. A genetic association study, including almost 50,000 T2D cases, found higher genetically-predicted BCAA concentrations were associated with increased T2D risk, suggesting a causal relationship^21^. A separate study, using genetic variants associated with BCAA and with insulin resistance, suggested insulin resistance leads to higher circulating BCAA concentrations, rather than the converse^22^. In combination, these findings suggest insulin resistance increases BCAA concentrations, which precede and contribute to T2D. This is consistent with persistence of the associations of BCAA in the present study after exclusion of T2D cases diagnosed during the first years of follow-up, and with previous descriptions of the trajectory from normoglycaemia to T2D^23^, highlighting a potentially valuable role for BCAA as markers of future T2D risk.

Our study showed strong inverse associations of omega-3 fatty acids with T2D risk. A large individual participant data meta-analysis, based on ~ 65,000 participants from 20 prospective studies (of mainly European ancestry) and > 16,000 cases of incident T2D, found qualitatively similar associations^24^. When analyses were limited to circulating fatty acids, individuals with combined omega-3 fatty acid, or docosahexaenoic acid, concentrations in the top, compared with the bottom, quintile had 23% and 24%, respectively, lower T2D risk. Prior investigations of the associations of fatty acids with T2D in Chinese populations are limited, but the described meta-analysis showed no clear heterogeneity across populations^24^. Although there are plausible mechanisms to support a protective effect of omega-3 fatty acids^24^, the causal relevance of the observed associations remains uncertain. However, the potential to influence omega-3 fatty acid levels through dietary intervention highlights the need for further investigation.

The large number of significant independent associations observed between circulating metabolic biomarkers and incident T2D risk in the present study in part reflects the focus of the metabolomics platform on lipid and lipoprotein measures, and correlations between these. The present study provides, for the first time, detailed characterisation of the relevance of lipoprotein size and subclass particle concentrations to T2D risk in a Chinese population. As shown in previous Western population studies^5,25^, we observed higher T2D risk among participants with higher concentrations of large VLDL particles and lower concentrations of large HDL particles, smaller mean HDL particle size and large mean VLDL particle size, as well as higher TG levels and lower HDL-cholesterol levels. This is consistent with an insulin resistant state^26^, which is a well-established component of the causal relationship between adiposity and T2D^27^. The observed stronger associations of certain metabolic biomarkers with T2D risk among centrally obese CKB participants may reflect greater prominence of insulin resistance in T2D aetiology among this population subgroup^22,26,28,29^. Although similar heterogeneity was not observed across BMI strata, the relative leanness of the study population prevented separate examination of the associations of metabolic biomarkers among participants with general obesity (i.e., BMI ≥ 30 kg/m^2^, observed in ~ 4% of the total CKB population^30^). At the same time, however, the population’s leanness provides a unique opportunity to expand our understanding of the aetiology of T2D among less adipose individuals and populations. In so doing, it valuably demonstrates the relevance of insulin resistance throughout the full adiposity range.

Recent prospective analyses among ~ 65,000 UK Biobank (UKB) participants examined the associations of 139 of the biomarkers considered herein (measured using the same NMR-metabolomics platform) with incident T2D (n = 1719) recorded during almost 12 years’ follow-up, adjusting for sociodemographic factors, fasting time, smoking, alcohol drinking and general and central adiposity^25^. Overall, the associations of 98 biomarkers were qualitatively consistent in the two study populations, including significant positive associations of 53 biomarkers with T2D risk and inverse associations of 27 biomarkers. However, the observed associations of several biomarkers appear more extreme in the CKB population, including BCAA (e.g., leucine HR 1.82 vs. 1.19 and valine 2.05 vs. 1.31 per 1 SD increment), apolipoprotein B/apolipoprotein A1 (1.79 vs. 1.09), and relative omega-3 fatty acid concentration (0.72 vs. 0.92). This is perhaps unexpected given the higher mean BMI in UKB (26.9 kg/m^2^) than in CKB (23.9 kg/m^2^ in subcohort participants). It is possible that these differences in the strength of the associations reflect, in part, ethnic differences in the typical pathophysiology of T2D^19^. Further studies directly comparing associations of metabolic biomarkers with T2D between ethnically diverse populations are needed, and may reveal novel insights into T2D aetiology.

The ability to identify individuals at greatest risk of T2D is vital for appropriate targeting of preventative interventions. Advances in “omics” research have stimulated interest in their potential for improving prediction of T2D risk over and above the traditional risk prediction models which frequently over-estimate actual risk^31^. An established risk prediction model in Chinese adults^20^ showed good discriminatory ability in CKB, with a c-statistic of 0.86, better than in the population in which it was developed (c-statistic 0.77^20^) and comparable to the performance of established models in other populations^31^. This strong discriminatory ability of established T2D risk prediction models presents challenges in identifying biomarkers capable of improving risk prediction. Thus, while addition of selected circulating metabolic biomarkers to the traditional T2D risk prediction model further improved its performance (c-statistic 0.91), the improvement was modest. Of note, however, although previous studies of mostly Western populations have observed enhanced discriminatory ability of T2D risk prediction models after inclusion of metabolic biomarkers, the degree of improvement was generally less marked^5,7,11,12,15,32^, with unclear generalisability to other populations. The few studies in China that have assessed this have frequently included limited biomarkers (e.g., restricted to amino acids^33^ or lipids^34^). The present study highlights the potential relevance of including biomarkers from diverse molecular pathways for improved risk prediction. Moreover, the standardised, targeted, high-throughput metabolomics platform used^35,36^ highlights the translational potential of the current study findings to clinical settings.

Our study had several strengths. It is among the largest Chinese population studies investigating prospective associations of circulating metabolic biomarkers with incident T2D^12,32–34,37,38^, and the largest to simultaneously investigate biomarkers across multiple diverse molecular pathways. Moreover, we employed an established targeted and validated metabolomics platform^39,40^, quantifying biomarker concentrations and enabling direct comparison with other studies. Furthermore, limited use of lipid-lowering medications in the study population reduced potential biases. However, the study had limitations. First, incident T2D was limited to diagnosed cases, although any associated misclassification would be expected to result in underestimation of associations of biomarkers with T2D. Second, repeat biomarker measurements were not available, preventing adjustment for intra-individual variation, again likely underestimating the strength of associations. Third, use of non-fasting blood samples may have increased inter-individual variation in biomarker concentrations. However, the analyses were adjusted for fasting time, as well as dietary factors, and there was no clear heterogeneity in associations across fasting time strata (data not shown). Fourth, lack of external validation of the risk prediction model incorporating metabolic biomarkers may have resulted in over-estimation of the model’s discriminatory ability. Finally, the observational nature of the study precludes conclusions regarding causality of observed associations.

Overall, the present study demonstrates highly significant associations of multiple circulating metabolic biomarkers from diverse molecular pathways with risk of future T2D in a relatively lean Chinese adult population. It highlights the ability of high-throughput, comprehensive, targeted NMR-metabolomic profiling to improve prediction of T2D beyond established risk factors (including glycaemia), demonstrating the potential clinical value of this approach in identifying those individuals most likely to benefit from early targeted T2D prevention efforts. Understanding of these associations is arguably of particular importance in China, where diabetes prevalence has escalated rapidly over recent decades, and continues to rise^2^.

## Methods

### Study population

Details of the CKB methods and population have been described previously^30^. Briefly, between June 2004 and July 2008, all permanent, non-disabled residents aged 35–74 years from 100 to 150 rural villages or urban committees in 10 study areas (5 urban and 5 rural) were invited to participate. Study areas were selected from China’s nationally representative Disease Surveillance Points. The overall response rate was ~ 30%, and 512,715 individuals were enrolled, including ~ 13,000 slightly outside the target age range (extending the participant age range to 30–79 years).

At baseline survey (and subsequent periodic resurveys of a random subset), participants completed laptop-based questionnaires administered by trained health workers, collecting information on demographic and lifestyle factors, and personal and family medical history. Physical measurements were collected using calibrated instruments by trained staff and included height, weight, WC, hip circumference, blood pressure and resting heart rate. A non-fasting venous blood sample was collected into an EDTA vacutainer (with hours since last meal recorded) and separated into one buffy coat and three plasma aliquots for long-term storage. Immediate on-site testing of RPG levels was undertaken using the SureStep Plus system (LifeScan, Milpitas, CA, USA). Participants with RPG ≥ 7.8 mmol/L and < 11.1 mmol/L were invited to return the following day for fasting plasma glucose measurement.

Participants were followed-up for cause-specific morbidity and mortality by electronic linkage, via unique national identification number, to disease (including diabetes) registries, death registries (ICD-10 coded by trained staff blinded to baseline information), and the national health insurance system (> 98% coverage across study areas) which provided ICD-10 coded diagnoses for all hospitalisations and deaths.

Ethics approval was obtained from the Oxford University Tropical Research Ethics Committee, the Chinese Center for Disease Control and Prevention Ethical Review Committee, and the Chinese Academy of Medical Sciences/Peking Union Medical College Ethical Committee. The CKB complies with all required ethical standards, guidelines and regulations for medical research on human subjects. All participants provided informed written consent.

### Case-cohort study

This case-cohort study^41^ included 900 participants with T2D, selected through simple random sampling from 7721 incident T2D cases (ICD10 E11) recorded during follow-up until 1 January 2017 (mean [SD] 7.9 [3.2] years). These cases were selected after excluding participants with self-reported or screen-detected (defined based on plasma glucose concentration and fasting time^42^) diabetes at baseline (n = 30,300) or without available plasma samples (n = 198). A subcohort of 905 was randomly selected from a sample of 31,443 participants selected at random from a subset of approximately 105,000 CKB cohort participants for whom genome-wide genotyping has been conducted^43^. Following exclusion of participants with inadequate plasma samples and mismatch of case status, as well as subcohort participants with self-reported or screen-detected diabetes at baseline, 882 T2D cases and a subcohort of 789 (of whom 26 were also included in the diabetes cases, consistent with the case-cohort design) were included in the main analyses.

### Metabolic biomarker quantification

Metabolomic profiling of T2D case and subcohort baseline plasma samples was undertaken using a high-throughput targeted NMR-metabolomics platform^35,36,39,44^, simultaneously profiling lipoprotein subclass distribution, particle size and composition, and quantifying lipids, fatty acids, amino acids, ketone bodies and other low molecular weight metabolic biomarkers. Overall, data were generated on 225 directly measured metabolic biomarkers (n = 146) or derived ratios (n = 79) of these biomarkers (Supplementary Table S1).

### Statistical analysis

Principal component analysis was used to detect individuals with extreme values; no exclusions were made after inspection of scatterplots of pairs of the first five principal components. Histograms were plotted to visually inspect metabolic biomarker distributions. The prevalence and mean values of baseline characteristics were calculated among T2D cases and the subcohort. Correlations between metabolic biomarkers among participants in the subcohort were assessed using Pearson partial correlation coefficients, adjusting for age, sex and study area.

Cox proportional hazards models fitted using the Prentice pseudo-partial likelihood (to account for the case-cohort study design)^41^ were used to estimate hazard ratios (HRs) for the associations of metabolic biomarkers with incident T2D, with time in study as the time scale. Models were adjusted for age (numeric), sex, study area (10 areas), education (6 categories), fasting time (numeric), smoking (ever regular vs. other), alcohol drinking (ever regular vs. other), physical activity (metabolic equivalent of task hours per day, numeric), dietary factors (frequency of consumption of meat, fish, fresh fruit, dairy products; 4 times/week or more vs. other), family history of diabetes (any first degree relative vs. none), BMI (numeric) and WC (numeric). Additional analyses further adjusted for plasma glucose quantified on the NMR-metabolomics platform. Each metabolic biomarker was examined as a categorical variable (divided into quartiles) to assess the shape of the associations. Metabolic biomarkers were also examined as continuous variables to estimate HR per 1-SD increment. No transformations were applied as the associations of most metabolic biomarkers were broadly consistent with a log-linear form.

The proportional hazards assumption was assessed using Schoenfeld residuals. FDR correction was used to account for multiple testing and the large number of highly correlated metabolic biomarkers^45^. Adjusted HRs per 1-SD higher metabolic biomarker were examined in population subgroups defined by age (30–54/55–79 years), sex, region and adiposity (BMI < 25.0/ ≥ 25.0 kg/m^2^^46^; WC < 90/ ≥ 90 cm in men, < 80/ ≥ 80 in women^47^). In sensitivity analyses, the main Cox regression analyses were repeated after excluding the first 2 years of follow-up to minimise reverse causality. Adjusted log HRs per 1 SD higher metabolic biomarker were plotted against differences in BMI associated with the same increment in the metabolic biomarker overall, and in adiposity-based population subgroups.

To assess whether circulating metabolic biomarkers could improve T2D risk discrimination, we added a group of selected biomarkers to an established T2D risk prediction model developed in a Chinese population^20^. This conventional model, including age, sex, study area, fasting time, BMI, family history of diabetes, education, blood pressure, resting heart rate, plasma glucose, triglycerides and statin use, was selected since, compared with other models, it was developed in a larger study population and the variables included more closely matched data available in CKB (Supplementary Table S2). Additional metabolic biomarkers were selected for inclusion in the novel risk prediction model using the approach of Cox and Battey^48^. The 225 metabolic biomarkers were laid on a 5 × 5 × 9 cuboid, and a Cox regression model was fitted with each set of explanatory variables indexed by each dimension of the cuboid, adjusting for variables included in the traditional model. The biomarkers most highly associated with T2D risk (defined as those with z > 2) were kept from each regression, and biomarkers identified as such on three occasions were selected for inclusion in the model. Among those, pairs of variables with correlation > 0.95 were identified and the second of each pair removed. The discriminatory ability of the two models (i.e., one with and one without metabolic biomarkers) was assessed and compared using a weighted C-index^49^.

All analyses were conducted using R version 4.0.5 (R Project for Statistical Computing, Vienna, Austria).

## Supplementary Information

Supplementary Information.

## Data Availability

The CKB is a global resource for the investigation of lifestyle, environmental, blood biochemical and genetic factors as determinants of common diseases. The CKB study group is committed to making the cohort data available to the scientific community in China, the UK and worldwide to advance knowledge about the causes, prevention and treatment of disease. For detailed information on what data are currently available to open access users and how to apply for it, visit: http://www.ckbiobank.org/site/Data+Access. Researchers who are interested in obtaining the raw data from the CKB study that underlies this paper should contact ckbaccess@ndph.ox.ac.uk. A research proposal will be requested to ensure that any analysis is performed by bona fide researchers and - where data is not currently available to open access researchers - is restricted to the topic covered in this paper.
