# Supplementary material for: The role of NMR-based circulating metabolic biomarkers in development and risk prediction of new onset type 2 diabetes
Source: Sci Rep. 2022 Sep 5;12:15071. doi: 10.1038/s41598-022-19159-8 (PMC9445062; doi:10.1038/s41598-022-19159-8)

This page is intentionally left blank

The role of NMR-based circulating metabolic biomarkers in development and risk prediction of new onset type 2 diabetes

Supplementary Materials, Table of Contents

Page 3. China Kadoorie Biobank collaborative group and members

Page 4. Supplementary Table S1. Associations of metabolic biomarkers with risk of incident type 2 diabetes

Page 8. Supplementary Table S2. Chinese population type 2 diabetes risk prediction models

Page 9. Supplementary Figure S1. Correlation between directly-measured metabolic biomarkers within subcohort participants (n=789)

Page 10. Supplementary Figure S2. Associations of directly-measured metabolic biomarkers with incident type 2 diabetes

Page 11. Supplementary Figure S3. Associations of metabolic biomarkers with incident type 2 diabetes

Page 13. Supplementary Figure S4. Associations of lipids, apolipoproteins and lipoprotein particle concentrations with risk of incident type 2 diabetes, stratified by adiposity

Page 14. Supplementary Figure S5. Associations of fatty acids, amino acids, glycolysis metabolites, ketone bodies and inflammatory markers with risk of incident type 2 diabetes, stratified by adiposity

Page 15. Supplementary Figure S6. Associations of lipids, apolipoproteins and lipoprotein particle concentrations with risk of incident type 2 diabetes, stratified by age

Page 16. Supplementary Figure S7. Associations of fatty acids, amino acids, glycolysis metabolites, ketone bodies and inflammatory markers with risk of incident type 2 diabetes, stratified by age

Page 17. Supplementary Figure S8. Associations of lipids, apolipoproteins and lipoprotein particle concentrations with risk of incident type 2 diabetes, stratified by sex

Page 18. Supplementary Figure S9. Associations of fatty acids, amino acids, glycolysis metabolites, ketone bodies and inflammatory markers with risk of incident type 2 diabetes, stratified by sex

Page 19. Supplementary Figure S10. Associations of lipids, apolipoproteins and lipoprotein particle concentrations with risk of incident type 2 diabetes, stratified by region

Page 20. Supplementary Figure S11. Associations of fatty acids, amino acids, glycolysis metabolites, ketone bodies and inflammatory markers with risk of incident type 2 diabetes, stratified by region

Page 21. Supplementary Figure S12. Associations of metabolic biomarkers with risk of incident type 2 diabetes (n=757) excluding the first 2 years of follow-up

China Kadoorie Biobank collaborative group and members

International Steering Committee: Junshi Chen, Zhengming Chen (PI), Robert Clarke, Rory Collins, Yu Guo, Liming Li (PI), Chen Wang, Jun Lv, Richard Peto, Robin Walters

International Co-ordinating Centre, Oxford: Daniel Avery, Fiona Bragg, Derrick Bennett, Ruth Boxall, Ka Hung Chan, Yumei Chang, Yiping Chen, Zhengming Chen, Johnathan Clarke; Robert Clarke, Huaidong Du, Zammy Fairhurst-Hunter, Hannah Fry, Wei Gan, Simon Gilbert, Alex Hacker, Parisa Hariri, Mike Hill, Michael Holmes, Pek Kei Im, Andri Iona, Maria Kakkoura, Christiana Kartsonaki, Rene Kerosi, Kuang Lin, Mohsen Mazidi, Iona Millwood, Qunhua Nie, Alfred Pozarickij, Paul Ryder, Sam Sansome, Dan Schmidt, Paul Sherliker, Rajani Sohoni, Becky Stevens, Iain Turnbull, Robin Walters, Lin Wang, Neil Wright, Ling Yang, Xiaoming Yang, Pang Yao

National Co-ordinating Centre, Beijing: Yu Guo, Xiao Han, Can Hou, Chun Li, Chao Liu, Jun Lv, Pei Pei, Canqing Yu

10 Regional Co-ordinating Centres:

Guangxi Provincial CDC: Naying Chen, Duo Liu, Zhenzhu Tang. Liuzhou CDC: Ningyu Chen, Qilian Jiang, Jian Lan, Mingqiang Li, Yun Liu, Fanwen Meng, Jinhuai Meng, Rong Pan, Yulu Qin, Ping Wang, Sisi Wang, Liuping Wei, Liyuan Zhou. **Gansu** Provincial CDC: Caixia Dong, Pengfei Ge, Xiaolan Ren. Maiji CDC: Zhongxiao Li, Enke Mao, Tao Wang, Hui Zhang, Xi Zhang. **Hainan** Provincial CDC: Jinyan Chen, Ximin Hu, Xiaohuan Wang. Meilan CDC: Zhendong Guo, Huimei Li, Yilei Li, Min Weng, Shukuan Wu. **Heilongjiang** Provincial CDC: Shichun Yan, Mingyuan Zou, Xue Zhou. Nangang CDC: Ziyan Guo, Quan Kang, Yanjie Li, Bo Yu, Qinai Xu. **Henan** Provincial CDC: Liang Chang, Lei Fan, Shixian Feng, Ding Zhang, Gang Zhou. Huixian CDC: Yulian Gao, Tianyou He, Pan He, Chen Hu, Huarong Sun, Xukui Zhang. **Hunan** Provincial CDC: Biyun Chen, Zhongxi Fu, Yuelong Huang, Huilin Liu, Qiaohua Xu, Li Yin. Liuyang CDC: Huajun Long, Xin Xu, Hao Zhang, Libo Zhang. **Jiangsu** Provincial CDC: Jian Su, Ran Tao, Ming Wu, Jie Yang, Jinyi Zhou, Yonglin Zhou. Suzhou CDC: Yihe Hu, Yujie Hua, Jianrong Jin Fang Liu, Jingchao Liu, Yan Lu, Liangcai Ma, Aiyu Tang, Jun Zhang. **Qingdao** Qingdao CDC: Liang Cheng, Ranran Du, Ruqin Gao, Feifei Li, Shanpeng Li, Yongmei Liu, Feng Ning, Zengchang Pang, Xiaohui Sun, Xiaocao Tian, Shaojie Wang, Yaoming Zhai, Hua Zhang, Licang CDC: Wei Hou, Silu Lv, Junzheng Wang. **Sichuan** Provincial CDC: Xiaofang Chen, Xianping Wu, Ningmei Zhang, Weiwei Zhou. Pengzhou CDC: Xiaofang Chen, Jianguo Li, Jiaqiu Liu, Guojin Luo, Qiang Sun, Xunfu Zhong. **Zhejiang** Provincial CDC: Weiwei Gong, Ruying Hu, Hao Wang, Meng Wan, Min Yu. Tongxiang CDC: Lingli Chen, Qijun Gu, Dongxia Pan, Chunmei Wang, Kaixu Xie, Xiaoyi Zhang

Supplementary Table S1. Associations of metabolic biomarkers with risk of incident type 2 diabetes

Metabolic biomarker	Mean (SD)	Model A		Model B		Model C1		Model C2		Model C3		Model D	
		HR* (95% CI)	*p*-value†	HR* (95% CI)	*p*-value†	HR* (95% CI)	*p*-value†	HR* (95% CI)	*p*-value†	HR* (95% CI)	*p*-value†	HR* (95% CI)	*p*-value†
18:2, linoleic acid	2.70 (0.56) mmol/L	1.88 (1.60, 2.21)	5.50×10^{-14}	1.87 (1.58, 2.21)	4.38×10^{-13}	1.74 (1.45, 2.09)	6.25×10^{-09}	1.72 (1.43, 2.07)	2.72×10^{-08}	1.72 (1.43, 2.07)	2.79×10^{-08}	1.64 (1.35, 1.99)	1.76×10^{-06}
22:6, docosahexaenoic acid	0.13 (0.048) mmol/L	0.79 (0.69, 0.90)	6.60×10^{-04}	0.78 (0.68, 0.89)	$4.00x10^{-04}$	0.68 (0.58, 0.81)	1.53×10^{-05}	0.66 (0.55, 0.79)	1.01×10^{-05}	0.66 (0.55, 0.79)	1.01×10^{-05}	0.67 (0.55, 0.82)	1.60×10^{-04}
3-hydroxybutyrate	0.13 (0.05) mmol/L	1.16 (1.01, 1.33)	0.05	1.17 (1.02, 1.35)	0.03	1.21 (1.05, 1.39)	0.01	1.21 (1.05, 1.39)	0.01	1.21 (1.05, 1.39)	0.01	1.32 (1.15, 1.51)	1.20×10^{-04}
Acetate	0.046 (0.079) mmol/L	1.11 (1.00, 1.22)	0.05	1.11 (1.01, 1.22)	0.04	1.06 (0.94, 1.20)	0.35	1.08 (0.95, 1.23)	0.29	1.08 (0.95, 1.23)	0.30	1.01 (0.89, 1.14)	0.94
Acetoacetate	0.027 (0.014) mmol/L	1.57 (1.31, 1.89)	2.28×10^{-06}	1.56 (1.29, 1.88)	7.75×10^{-06}	1.34 (1.16, 1.54)	1.10×10^{-04}	1.31 (1.14, 1.51)	$4.00x10^{-04}$	1.31 (1.14, 1.51)	4.20×10^{-04}	1.32 (1.10, 1.58)	5.00×10^{-03}
Alanine	0.39 (0.062) mmol/L	1.69 (1.47, 1.94)	4.50×10^{-13}	1.70 (1.48, 1.95)	3.17×10^{-13}	1.58 (1.35, 1.84)	2.09×10^{-08}	1.59 (1.35, 1.87)	6.19×10^{-08}	1.59 (1.35, 1.87)	6.43×10^{-08}	1.38 (1.15, 1.66)	1.00×10^{-03}
Albumin	0.086 (0.0035) signal area	1.11 (0.98, 1.26)	0.12	1.11 (0.98, 1.27)	0.13	1.02 (0.88, 1.18)	0.78	0.98 (0.84, 1.14)	0.79	0.98 (0.84, 1.14)	0.80	0.97 (0.82, 1.16)	0.78
Apolipoprotein A-I	1.40 (0.15) g/L	0.67 (0.59, 0.76)	4.15×10^{-09}	0.66 (0.57, 0.75)	2.82×10^{-09}	0.64 (0.54, 0.75)	1.26×10^{-07}	0.62 (0.52, 0.73)	3.65×10^{-08}	0.62 (0.52, 0.73)	3.43×10^{-08}	0.57 (0.47, 0.68)	1.02×10^{-08}
Apolipoprotein B	0.77 (0.16) g/L	1.48 (1.28, 1.71)	1.74×10^{-07}	1.46 (1.26, 1.70)	6.81×10^{-07}	1.32 (1.12, 1.55)	1.30×10^{-03}	1.28 (1.09, 1.50)	5.00×10^{-03}	1.28 (1.09, 1.50)	5.00×10^{-03}	1.29 (1.08, 1.54)	7.70×10^{-03}
CE in chylomicrons and extremely large VLDL	0.0032 (0.0023) mmol/L	1.85 (1.57, 2.17)	6.91×10^{-13}	1.90 (1.62, 2.23)	1.16×10^{-14}	1.64 (1.38, 1.95)	3.86×10^{-08}	1.61 (1.36, 1.91)	1.53×10^{-07}	1.61 (1.36, 1.91)	1.51×10^{-07}	1.59 (1.31, 1.93)	5.44×10^{-06}
CE in IDL	0.38 (0.10) mmol/L	0.89 (0.77, 1.02)	0.12	0.88 (0.76, 1.02)	0.1	0.86 (0.73, 1.02)	0.12	0.83 (0.70, 0.99)	0.05	0.83 (0.70, 0.99)	0.05	0.86 (0.72, 1.04)	0.16
CE in large HDL	0.24 (0.093) mmol/L	0.46 (0.38, 0.56)	2.55×10^{-14}	0.46 (0.38, 0.56)	1.38×10^{-14}	0.50 (0.41, 0.61)	9.41×10^{-11}	0.50 (0.40, 0.62)	5.99×10^{-10}	0.50 (0.40, 0.62)	6.34×10^{-10}	0.47 (0.37, 0.59)	2.29×10^{-09}
CE in large LDL	0.47 (0.14) mmol/L	0.95 (0.82, 1.10)	0.51	0.94 (0.81, 1.10)	0.47	0.94 (0.79, 1.12)	0.51	0.90 (0.75, 1.08)	0.29	0.90 (0.75, 1.08)	0.29	0.93 (0.78, 1.12)	0.5
CE in large VLDL	0.039 (0.023) mmol/L	1.93 (1.64, 2.26)	7.28×10^{-15}	1.98 (1.69, 2.32)	7.40×10^{-17}	1.71 (1.44, 2.02)	2.93×10^{-09}	1.68 (1.42, 1.99)	8.68×10^{-09}	1.68 (1.42, 2.00)	8.42×10^{-09}	1.69 (1.40, 2.04)	1.62×10^{-07}
CE in medium HDL	0.31 (0.057) mmol/L	0.83 (0.73, 0.95)	8.80×10^{-03}	0.81 (0.71, 0.93)	3.50×10^{-03}	0.79 (0.68, 0.91)	2.50×10^{-03}	0.77 (0.66, 0.90)	1.40×10^{-03}	0.77 (0.66, 0.90)	1.50×10^{-03}	0.69 (0.58, 0.83)	1.20×10^{-04}
CE in medium LDL	0.26 (0.099) mmol/L	0.94 (0.81, 1.10)	0.49	0.94 (0.81, 1.10)	0.47	0.95 (0.80, 1.13)	0.61	0.92 (0.77, 1.10)	0.38	0.92 (0.77, 1.10)	0.39	0.94 (0.79, 1.13)	0.58
CE in medium VLDL	0.09 (0.04) mmol/L	1.74 (1.49, 2.03)	4.58×10^{-12}	1.75 (1.50, 2.04)	3.91×10^{-12}	1.52 (1.29, 1.80)	1.95×10^{-06}	1.51 (1.28, 1.77)	2.76×10^{-06}	1.51 (1.28, 1.78)	2.64×10^{-06}	1.58 (1.32, 1.89)	1.77×10^{-06}
CE in small HDL	0.31 (0.054) mmol/L	0.78 (0.66, 0.92)	3.60×10^{-03}	0.77 (0.65, 0.91)	$2.60x10^{-03}$	0.81 (0.68, 0.97)	0.03	0.79 (0.66, 0.94)	0.01	0.79 (0.66, 0.94)	0.01	0.79 (0.65, 0.95)	0.02
CE in small LDL	0.16 (0.06) mmol/L	0.89 (0.77, 1.04)	0.16	0.89 (0.76, 1.04)	0.16	0.91 (0.76, 1.08)	0.31	0.87 (0.73, 1.05)	0.17	0.87 (0.73, 1.05)	0.17	0.91 (0.76, 1.08)	0.33
CE in small VLDL	0.11 (0.043) mmol/L	0.99 (0.87, 1.12)	0.85	0.98 (0.86, 1.11)	0.78	0.91 (0.79, 1.06)	0.28	0.88 (0.76, 1.02)	0.12	0.88 (0.76, 1.02)	0.12	0.95 (0.80, 1.12)	0.56
CE in very large HDL	0.14 (0.056) mmol/L	0.48 (0.41, 0.56)	5.72×10^{-18}	0.48 (0.41, 0.57)	1.93×10^{-17}	0.48 (0.40, 0.58)	3.30×10^{-13}	0.47 (0.39, 0.57)	1.49×10^{-13}	0.47 (0.39, 0.57)	1.21×10^{-13}	0.47 (0.38, 0.58)	6.28×10^{-12}
CE in very large VLDL	0.0094 (0.0063) mmol/L	1.82 (1.54, 2.15)	6.51×10^{-12}	1.89 (1.61, 2.22)	2.45×10^{-14}	1.63 (1.37, 1.94)	9.61×10^{-08}	1.59 (1.34, 1.89)	4.43×10^{-07}	1.60 (1.34, 1.90)	4.36×10^{-07}	1.54 (1.26, 1.87)	5.40×10^{-05}
CE in very small VLDL	0.13 (0.038) mmol/L	0.73 (0.64, 0.83)	5.55×10^{-06}	0.72 (0.63, 0.83)	4.84×10^{-06}	0.69 (0.59, 0.81)	1.81×10^{-05}	0.66 (0.56, 0.78)	2.76×10^{-06}	0.66 (0.56, 0.78)	2.64×10^{-06}	0.70 (0.58, 0.84)	2.20×10^{-04}
CE:TL in chylomicrons and extremely large VLDL	10.00 (3.70) %	0.76 (0.66, 0.88)	4.20×10^{-04}	0.77 (0.67, 0.90)	$8.40x10^{-04}$	0.82 (0.69, 0.97)	0.03	0.84 (0.71, 1.01)	0.08	0.84 (0.71, 1.01)	0.08	0.89 (0.73, 1.08)	0.30
CE:TL in IDL	44.00 (2.30) %	0.89 (0.79, 1.01)	0.09	0.89 (0.78, 1.01)	0.09	0.85 (0.73, 0.98)	0.03	0.81 (0.70, 0.94)	9.50×10^{-03}	0.81 (0.70, 0.94)	9.60×10^{-03}	0.81 (0.68, 0.96)	0.03
CE:TL in large HDL	37.00 (2.20) %	0.62 (0.54, 0.72)	8.34×10^{-10}	0.61 (0.53, 0.71)	2.38×10^{-10}	0.66 (0.56, 0.78)	8.74×10^{-07}	0.69 (0.58, 0.81)	1.91×10^{-05}	0.69 (0.58, 0.81)	1.71×10^{-05}	0.68 (0.57, 0.81)	2.52×10^{-05}
CE:TL in large LDL	46.00 (3.00) %	0.86 (0.73, 1.01)	0.09	0.87 (0.73, 1.03)	0.12	0.87 (0.72, 1.05)	0.17	0.83 (0.68, 1.00)	0.06	0.83 (0.68, 1.00)	0.06	0.84 (0.70, 1.02)	0.12
CE:TL in large VLDL	14.00 (2.10) %	0.63 (0.54, 0.73)	1.47×10^{-08}	0.62 (0.53, 0.73)	2.14×10^{-08}	0.75 (0.63, 0.91)	4.10×10^{-03}	0.80 (0.67, 0.96)	0.03	0.80 (0.67, 0.96)	0.03	0.88 (0.72, 1.08)	0.27
CE:TL ratio in medium HDL	40.00 (2.50) %	0.71 (0.62, 0.82)	3.50×10^{-06}	0.71 (0.62, 0.81)	1.30×10^{-06}	0.75 (0.64, 0.87)	3.60×10^{-04}	0.75 (0.64, 0.88)	6.00×10^{-04}	0.75 (0.64, 0.88)	6.10×10^{-04}	0.64 (0.56, 0.73)	1.91×10^{-10}
CE:TL ratio in medium LDL	43.00 (6.90) %	0.80 (0.69, 0.92)	$2.60x10^{-03}$	0.80 (0.69, 0.93)	$5.70x10^{-03}$	0.84 (0.71, 0.99)	0.05	0.82 (0.70, 0.97)	0.03	0.82 (0.70, 0.97)	0.03	0.85 (0.72, 1.00)	0.08
CE:TL ratio in medium VLDL	16.00 (2.90) %	0.64 (0.55, 0.74)	3.08×10^{-09}	0.63 (0.54, 0.74)	8.59×10^{-09}	0.72 (0.61, 0.85)	1.50×10^{-04}	0.73 (0.62, 0.87)	7.70×10^{-04}	0.73 (0.62, 0.87)	7.90×10^{-04}	0.78 (0.65, 0.95)	0.02
CE:TL ratio in small HDL	30.00 (4.60) %	0.63 (0.53, 0.75)	4.31×10^{-07}	0.62 (0.52, 0.73)	5.42×10^{-08}	0.69 (0.57, 0.83)	2.10×10^{-04}	0.69 (0.57, 0.84)	2.40×10^{-04}	0.69 (0.57, 0.83)	2.40×10^{-04}	0.74 (0.60, 0.90)	4.90×10^{-03}
CE:TL ratio in small LDL	41.00 (7.30) %	0.77 (0.67, 0.88)	4.10×10^{-04}	0.77 (0.67, 0.90)	$9.50x10^{-04}$	0.82 (0.69, 0.96)	0.02	0.80 (0.68, 0.95)	0.01	0.80 (0.68, 0.95)	0.01	0.84 (0.71, 0.99)	0.06
CE:TL ratio in small VLDL	20.00 (5.20) %	0.58 (0.50, 0.68)	5.18×10^{-12}	0.56 (0.48, 0.66)	1.23×10^{-12}	0.59 (0.49, 0.70)	9.46×10^{-09}	0.57 (0.48, 0.69)	8.23×10^{-09}	0.57 (0.48, 0.69)	6.78×10^{-09}	0.58 (0.48, 0.70)	7.97×10^{-08}
CE:TL ratio in very large HDL	33.00 (4.80) %	1.06 (0.88, 1.27)	0.57	1.06 (0.88, 1.28)	0.56	0.93 (0.76, 1.14)	0.53	0.88 (0.72, 1.08)	0.25	0.88 (0.72, 1.08)	0.25	0.91 (0.73, 1.13)	0.45
CE:TL ratio in very large VLDL	13.00 (3.30) %	0.65 (0.55, 0.77)	2.08×10^{-06}	0.65 (0.54, 0.77)	1.95×10^{-06}	0.74 (0.62, 0.90)	3.40×10^{-03}	0.79 (0.65, 0.95)	0.02	0.79 (0.65, 0.95)	0.02	0.88 (0.74, 1.06)	0.22
CE:TL ratio in very small VLDL	32.00 (4.00) %	0.56 (0.46, 0.68)	1.05×10^{-08}	0.54 (0.46, 0.63)	4.70×10^{-14}	0.56 (0.47, 0.66)	1.13×10^{-10}	0.55 (0.46, 0.65)	8.93×10^{-11}	0.55 (0.46, 0.65)	8.02×10^{-11}	0.55 (0.46, 0.66)	1.49×10^{-09}
Citrate	0.15 (0.021) mmol/L	1.10 (0.98, 1.24)	0.14	1.10 (0.97, 1.25)	0.14	1.08 (0.94, 1.25)	0.29	1.12 (0.96, 1.29)	0.17	1.11 (0.96, 1.29)	0.17	1.05 (0.89, 1.24)	0.60
Chylomicrons and extremely large VLDL particles	$1.5e\times10^{-10}$ (1.1×10^{-10}) mol/L	2.05 (1.73, 2.44)	2.26×10^{-15}	2.19 (1.86, 2.58)	1.03×10^{-19}	1.87 (1.57, 2.24)	2.65×10^{-11}	1.81 (1.52, 2.17)	4.99×10^{-10}	1.82 (1.52, 2.17)	4.93×10^{-10}	1.68 (1.38, 2.06)	1.21×10^{-06}
IDL particles	8.6e-08 (2e-08) mol/L	0.95 (0.82, 1.10)	0.54	0.94 (0.81, 1.10)	0.47	0.94 (0.79, 1.12)	0.53	0.91 (0.77, 1.08)	0.34	0.91 (0.77, 1.08)	0.34	0.95 (0.79, 1.14)	0.61
Large HDL particles	1×10^{-6} ($\times10^{-7}$) mol/L	0.48 (0.39, 0.58)	9.74×10^{-14}	0.47 (0.39, 0.57)	4.72×10^{-14}	0.50 (0.41, 0.62)	9.79×10^{-11}	0.50 (0.41, 0.62)	5.03×10^{-10}	0.50 (0.41, 0.62)	5.30×10^{-10}	0.47 (0.37, 0.59)	1.16×10^{-09}
Large LDL particles	1.4×10^{-7} (3.5×10^{-8}) mol/L	0.99 (0.85, 1.15)	0.87	0.98 (0.84, 1.14)	0.79	0.98 (0.82, 1.16)	0.80	0.94 (0.79, 1.12)	0.53	0.94 (0.79, 1.12)	0.53	0.97 (0.81, 1.17)	0.79
Large VLDL particles	5.1×10^{-9} (3.4×10^{-9}) mol/L	1.97 (1.67, 2.31)	1.64×10^{-15}	2.05 (1.76, 2.39)	5.17×10^{-19}	1.75 (1.48, 2.07)	3.41×10^{-10}	1.70 (1.44, 2.02)	3.64×10^{-09}	1.71 (1.44, 2.02)	3.67×10^{-09}	1.65 (1.36, 1.99)	1.09×10^{-06}
Medium HDL particles	1.8×10^{-6} (2.9×10^{-7}) mol/L	1.02 (0.89, 1.17)	0.79	0.99 (0.86, 1.14)	0.90	0.92 (0.79, 1.07)	0.32	0.90 (0.77, 1.05)	0.22	0.90 (0.77, 1.05)	0.22	0.81 (0.68, 0.96)	0.03
Medium LDL particles	1.1×10^{-7} (3×10^{-8}) mol/L	1.08 (0.93, 1.24)	0.35	1.07 (0.92, 1.24)	0.43	1.06 (0.90, 1.26)	0.52	1.02 (0.86, 1.22)	0.81	1.02 (0.86, 1.22)	0.8	1.04 (0.87, 1.24)	0.72
Medium VLDL particles	1.8×10^{-8} (8.8×10^{-9}) mol/L	2.00 (1.71, 2.35)	2.02×10^{-16}	2.07 (1.77, 2.41)	3.69×10^{-19}	1.75 (1.48, 2.07)	3.22×10^{-10}	1.71 (1.44, 2.03)	3.15×10^{-09}	1.71 (1.45, 2.03)	2.93×10^{-09}	1.72 (1.42, 2.07)	7.80×10^{-08}
Small HDL particles	4.6×10^{-6} (4.3×10^{-7}) mol/L	1.47 (1.29, 1.68)	1.02×10^{-08}	1.46 (1.28, 1.66)	2.84×10^{-08}	1.29 (1.13, 1.49)	5.10×10^{-04}	1.24 (1.07, 1.43)	5.50×10^{-03}	1.24 (1.07, 1.43)	5.30×10^{-03}	1.14 (0.97, 1.34)	0.15
Small LDL particles	1.3×10^{-7} (3.3×10^{-8}) mol/L	1.07 (0.93, 1.23)	0.40	1.06 (0.91, 1.22)	0.48	1.04 (0.88, 1.23)	0.67	1.00 (0.84, 1.18)	0.98	1.00 (0.84, 1.19)	0.99	1.01 (0.85, 1.20)	0.92
Small VLDL particles	2.9×10^{-8} (9.5×10^{-9}) mol/L	1.82 (1.54, 2.14)	2.65×10^{-12}	1.84 (1.57, 2.15)	1.70×10^{-13}	1.59 (1.35, 1.89)	1.71×10^{-07}	1.58 (1.33, 1.86)	3.20×10^{-07}	1.58 (1.33, 1.86)	2.97×10^{-07}	1.63 (1.35, 1.95)	9.09×10^{-07}
Very large HDL particles	4.1×10^{-7} (1.6×10^{-7}) mol/L	0.45 (0.37, 0.53)	1.78×10^{-17}	0.44 (0.37, 0.53)	2.83×10^{-17}	0.46 (0.38, 0.57)	8.58×10^{-13}	0.45 (0.37, 0.56)	1.20×10^{-12}	0.45 (0.37, 0.56)	1.17×10^{-12}	0.45 (0.36, 0.57)	4.34×10^{-11}
Very large VLDL particles	8.1×10^{-10} (6×10^{-10}) mol/L	1.92 (1.62, 2.27)	9.58×10^{-14}	2.02 (1.72, 2.37)	2.13×10^{-17}	1.72 (1.45, 2.05)	3.45×10^{-09}	1.67 (1.40, 1.99)	3.81×10^{-08}	1.67 (1.40, 1.99)	3.76×10^{-08}	1.56 (1.27, 1.91)	3.78×10^{-05}
Very small VLDL particles	3.3×10^{-8} (7.6×10^{-9}) mol/L	1.15 (1.00, 1.31)	0.06	1.14 (0.99, 1.30)	0.09	1.07 (0.91, 1.24)	0.47	1.03 (0.88, 1.20)	0.75	1.03 (0.88, 1.20)	0.74	1.09 (0.91, 1.31)	0.40
Creatinine	0.054 (0.029) mmol/L	1.08 (1.01, 1.15)	0.03	1.09 (1.02, 1.16)	0.01	1.06 (0.99, 1.13)	0.12	1.08 (1.01, 1.16)	0.03	1.08 (1.01, 1.16)	0.03	1.07 (0.99, 1.14)	0.10
Esterified cholesterol	2.40 (0.48) mmol/L	1.20 (1.05, 1.38)	0.01	1.18 (1.03, 1.37)	0.03	1.20 (1.03, 1.41)	0.03	1.18 (1.00, 1.39)	0.06	1.18 (1.00, 1.39)	0.06	1.11 (0.94, 1.32)	0.29
Estimated degree of unsaturation	1.10 (0.06)	1.22 (1.09, 1.36)	7.40×10^{-04}	1.23 (1.10, 1.38)	4.80×10^{-04}	1.33 (1.17, 1.52)	4.35×10^{-05}	1.40 (1.22, 1.61)	4.05×10^{-06}	1.40 (1.22, 1.61)	4.06×10^{-06}	1.33 (1.14, 1.56)	7.20×10^{-04}
FC	1.10 (0.19) mmol/L	1.12 (0.97, 1.29)	0.14	1.11 (0.96, 1.28)	0.20	1.11 (0.94, 1.31)	0.27	1.09 (0.92, 1.29)	0.35	1.09 (0.92, 1.29)	0.35	1.07 (0.89, 1.29)	0.51

Metabolic biomarker	Mean (SD)	Model A		Model B		Model C1		Model C2		Model C3		Model D	
		HR* (95% CI)	*p*-value†	HR* (95% CI)	*p*-value†	HR* (95% CI)	*p*-value†	HR* (95% CI)	*p*-value†	HR* (95% CI)	*p*-value†	HR* (95% CI)	*p*-value†
FC in chylomicrons and extremely large VLDL	0.0022 (0.0018) mmol/L	1.98 (1.67, 2.35)	2.05×10^{-14}	2.10 (1.79, 2.46)	9.54×10^{-19}	1.79 (1.51, 2.14)	2.56×10^{-10}	1.74 (1.46, 2.07)	4.18×10^{-09}	1.74 (1.46, 2.07)	4.14×10^{-09}	1.62 (1.32, 1.98)	7.25×10^{-06}
FC in IDL	0.15 (0.042) mmol/L	0.69 (0.58, 0.81)	2.04×10−05	0.68 (0.57, 0.80)	1.36×10^{-05}	0.71 (0.59, 0.86)	8.20×10^{-04}	0.70 (0.57, 0.84)	4.40×10^{-04}	0.70 (0.57, 0.84)	4.40×10^{-04}	0.73 (0.60, 0.88)	2.80×10^{-03}
FC in large HDL	0.064 (0.029) mmol/L	0.40 (0.32, 0.50)	5.47×10^{-16}	0.40 (0.32, 0.49)	1.13×10^{-16}	0.43 (0.35, 0.53)	4.76×10^{-13}	0.43 (0.34, 0.54)	2.53×10^{-12}	0.43 (0.34, 0.54)	3.00×10^{-12}	0.40 (0.31, 0.52)	1.73×10^{-11}
FC in large LDL	0.19 (0.047) mmol/L	0.74 (0.63, 0.88)	6.00×10^{-04}	0.74 (0.63, 0.88)	7.10×10^{-04}	0.77 (0.64, 0.94)	0.01	0.75 (0.62, 0.91)	6.10×10^{-03}	0.75 (0.62, 0.91)	6.20×10^{-03}	0.80 (0.66, 0.97)	0.03
FC in large VLDL	0.028 (0.023) mmol/L	1.95 (1.66, 2.29)	2.80×10^{-15}	2.03 (1.74, 2.37)	9.72×10^{-19}	1.73 (1.46, 2.05)	6.82×10^{-10}	1.69 (1.42, 2.00)	6.45×10^{-09}	1.69 (1.42, 2.00)	6.64×10^{-09}	1.62 (1.34, 1.96)	2.56×10^{-06}
FC in medium HDL	0.064 (0.015) mmol/L	0.80 (0.70, 0.92)	3.20×10^{-03}	0.78 (0.67, 0.90)	8.50×10^{-04}	0.75 (0.64, 0.88)	8.20×10^{-04}	0.74 (0.63, 0.87)	4.60×10^{-04}	0.74 (0.63, 0.87)	4.60×10^{-04}	0.66 (0.55, 0.80)	3.40×10^{-05}
FC in medium LDL	0.11 (0.022) mmol/L	0.96 (0.83, 1.10)	0.57	0.95 (0.82, 1.10)	0.52	0.95 (0.80, 1.13)	0.59	0.91 (0.76, 1.09)	0.34	0.91 (0.77, 1.09)	0.35	0.93 (0.78, 1.11)	0.48
FC in medium VLDL	0.066 (0.037) mmol/L	1.98 (1.69, 2.33)	5.34×10^{-16}	2.04 (1.75, 2.38)	9.72×10^{-19}	1.73 (1.46, 2.05)	7.33×10^{-10}	1.69 (1.43, 2.00)	6.09×10^{-09}	1.69 (1.43, 2.01)	5.61×10^{-09}	1.70 (1.40, 2.05)	1.61×10^{-07}
FC in small HDL	0.10 (0.012) mmol/L	1.28 (1.13, 1.46)	2.70×10^{-04}	1.27 (1.11, 1.44)	6.70×10^{-04}	1.11 (0.96, 1.28)	0.19	1.07 (0.92, 1.24)	0.43	1.07 (0.92, 1.24)	0.43	0.96 (0.81, 1.14)	0.72
FC in small LDL	0.066 (0.013) mmol/L	0.94 (0.82, 1.08)	0.40	0.93 (0.81, 1.07)	0.36	0.92 (0.78, 1.09)	0.37	0.88 (0.74, 1.04)	0.17	0.88 (0.74, 1.04)	0.17	0.89 (0.75, 1.06)	0.24
FC in small VLDL	0.074 (0.024) mmol/L	1.56 (1.33, 1.82)	5.34×10^{-08}	1.56 (1.35, 1.82)	1.13×10^{-08}	1.39 (1.18, 1.63)	1.10×10^{-04}	1.37 (1.17, 1.60)	2.00×10^{-04}	1.37 (1.17, 1.60)	$2.00x10^{-04}$	1.42 (1.19, 1.69)	$2.00x10^{-04}$
FC in very large HDL	0.051 (0.023) mmol/L	0.46 (0.39, 0.54)	7.51×10^{-18}	0.46 (0.39, 0.55)	1.93×10^{-17}	0.48 (0.39, 0.58)	4.34×10^{-13}	0.47 (0.38, 0.57)	3.65×10^{-13}	0.47 (0.39, 0.57)	3.47×10^{-13}	0.47 (0.38, 0.58)	2.61×10^{-11}
FC in very large VLDL	0.0066 (0.0055) mmol/L	1.86 (1.58, 2.20)	8.15×10^{-13}	1.94 (1.66, 2.28)	1.24×10^{-15}	1.65 (1.39, 1.97)	3.86×10^{-08}	1.61 (1.35, 1.91)	3.16×10^{-07}	1.61 (1.35, 1.92)	3.09×10^{-07}	1.50 (1.23, 1.84)	1.70×10^{-04}
FC in very small VLDL	0.061 (0.017) mmol/L	0.77 (0.66, 0.89)	1.10×10^{-03}	0.76 (0.65, 0.89)	7.80×10^{-04}	0.74 (0.62, 0.89)	1.90×10^{-03}	0.71 (0.60, 0.86)	6.10×10^{-04}	0.72 (0.60, 0.86)	6.20×10^{-04}	0.75 (0.63, 0.91)	5.50×10^{-03}
FC:TL in chylomicrons and extremely large VLDL	6.10 (2.00) %	1.14 (1.02, 1.26)	0.02	1.14 (1.03, 1.27)	0.02	1.10 (1.00, 1.22)	0.08	1.11 (1.00, 1.23)	0.06	1.11 (1.00, 1.23)	0.06	1.10 (0.98, 1.24)	0.13
FC:TL in IDL	17.00 (1.80) %	0.57 (0.47, 0.70)	4.28×10^{-08}	0.54 (0.45, 0.66)	9.10×10^{-10}	0.59 (0.48, 0.72)	8.74×10^{-07}	0.59 (0.48, 0.73)	1.35×10^{-06}	0.59 (0.48, 0.73)	1.36×10^{-06}	0.58 (0.49, 0.71)	7.97×10^{-08}
FC:TL in large HDL	9.60 (1.50) %	0.44 (0.36, 0.54)	1.64×10^{-15}	0.42 (0.35, 0.51)	2.81×10^{-17}	0.44 (0.36, 0.53)	1.07×10^{-15}	0.45 (0.37, 0.54)	6.73×10^{-14}	0.45 (0.37, 0.54)	3.75×10^{-14}	0.43 (0.35, 0.53)	1.40×10^{-14}
FC:TL in large LDL	19.00 (1.20) %	0.46 (0.38, 0.56)	1.19×10^{-13}	0.43 (0.36, 0.52)	1.73×10^{-19}	0.49 (0.41, 0.60)	5.04×10^{-12}	0.51 (0.42, 0.62)	1.00×10^{-10}	0.51 (0.42, 0.62)	1.07×10^{-10}	0.53 (0.43, 0.66)	1.96×10^{-08}
FC:TL in large VLDL	8.50 (2.20) %	1.90 (1.60, 2.26)	9.92×10^{-13}	1.89 (1.59, 2.24)	9.27×10^{-13}	1.58 (1.32, 1.89)	1.50×10^{-06}	1.51 (1.27, 1.81)	1.12×10^{-05}	1.51 (1.27, 1.81)	1.19×10^{-05}	1.42 (1.18, 1.73)	5.70×10^{-04}
FC:TL in medium HDL	8.20 (0.66) %	0.56 (0.48, 0.66)	2.93×10^{-12}	0.54 (0.46, 0.63)	1.08×10^{-14}	0.55 (0.47, 0.65)	2.48×10^{-11}	0.54 (0.45, 0.64)	2.35×10^{-11}	0.54 (0.45, 0.64)	2.51×10^{-11}	0.49 (0.40, 0.59)	1.00×10^{-12}
FC:TL in medium LDL	20.00 (1.90) %	0.85 (0.72, 1.00)	0.06	0.85 (0.72, 1.00)	0.06	0.87 (0.73, 1.03)	0.13	0.89 (0.76, 1.06)	0.23	0.89 (0.75, 1.06)	0.23	0.87 (0.72, 1.05)	0.18
FC:TL in medium VLDL	11.00 (1.00) %	1.87 (1.57, 2.22)	6.54×10^{-12}	1.84 (1.56, 2.19)	4.72×10^{-12}	1.53 (1.28, 1.84)	1.08×10^{-05}	1.46 (1.22, 1.76)	1.20×10^{-04}	1.46 (1.22, 1.76)	1.20×10^{-04}	1.48 (1.20, 1.82)	5.70×10^{-04}
FC:TL in small HDL	9.80 (0.55) %	0.90 (0.77, 1.05)	0.20	0.89 (0.76, 1.04)	0.16	0.81 (0.68, 0.97)	0.03	0.82 (0.68, 0.98)	0.04	0.82 (0.68, 0.97)	0.03	0.76 (0.63, 0.93)	0.01
FC:TL in small LDL	18.00 (1.40) %	0.80 (0.68, 0.94)	9.40×10^{-03}	0.80 (0.68, 0.94)	9.10×10^{-03}	0.83 (0.70, 0.98)	0.04	0.85 (0.72, 1.00)	0.07	0.85 (0.72, 1.00)	0.07	0.81 (0.67, 0.98)	0.05
FC:TL in small VLDL	13.00 (0.67) %	0.62 (0.54, 0.72)	1.23×10^{-10}	0.60 (0.52, 0.70)	2.26×10^{-11}	0.62 (0.52, 0.73)	2.24×10^{-08}	0.60 (0.50, 0.71)	2.72×10^{-08}	0.60 (0.50, 0.71)	2.79×10^{-08}	0.60 (0.50, 0.72)	7.39×10^{-08}
FC:TL in very large HDL	12.00 (1.30) %	0.69 (0.59, 0.81)	7.52×10^{-06}	0.69 (0.59, 0.81)	7.75×10^{-06}	0.71 (0.61, 0.83)	3.51×10^{-05}	0.70 (0.60, 0.82)	$1.83\times10^{-0}5$	0.70 (0.60, 0.82)	1.82×10^{-05}	0.70 (0.60, 0.81)	4.86×10^{-06}
FC:TL in very large VLDL	7.50 (2.00) %	1.35 (1.19, 1.55)	1.21×10^{-05}	1.34 (1.17, 1.54)	6.19×10^{-05}	1.24 (1.04, 1.49)	0.03	1.23 (1.03, 1.47)	0.03	1.23 (1.03, 1.48)	0.03	1.10 (0.93, 1.31)	0.30
FC:TL in very small VLDL	15.00 (1.40) %	0.66 (0.55, 0.80)	2.84×10^{-05}	0.64 (0.53, 0.78)	1.03×10^{-05}	0.67 (0.55, 0.82)	1.60×10^{-04}	0.68 (0.56, 0.82)	1.60×10^{-04}	0.67 (0.56, 0.82)	1.60×10^{-04}	0.64 (0.53, 0.76)	2.89×10^{-06}
Glucose	4.10 (1.90) mmol/L	3.45 (2.67, 4.46)	2.60×10^{-19}	3.64 (2.82, 4.69)	1.70×10^{-21}	3.67 (2.82, 4.78)	1.27×10^{-19}	3.49 (2.70, 4.52)	6.57×10^{-19}	3.53 (2.72, 4.58)	7.14×10^{-19}	N/A	N/A
Glutamine	0.46 (0.066) mmol/L	1.15 (1.01, 1.31)	0.05	1.18 (1.04, 1.35)	0.02	1.21 (1.05, 1.40)	0.02	1.22 (1.05, 1.43)	0.04	1.22 (1.05, 1.42)	0.01	1.17 (0.98, 1.40)	0.12
Glycoprotein acetyls, mainly a1-acid glycoprotein	1.20 (0.24) mmol/L	1.20 (1.06, 1.35)	5.80×10^{-03}	1.20 (1.06, 1.36)	6.70×10^{-03}	1.00 (0.87, 1.16)	0.99	0.95 (0.81, 1.11)	0.53	0.95 (0.81, 1.11)	0.53	0.94 (0.79, 1.11)	0.53
Histidine	0.058 (0.006) mmol/L	1.10 (0.97, 1.25)	0.15	1.12 (0.98, 1.27)	0.1	1.11 (0.96, 1.29)	0.19	1.12 (0.96, 1.31)	0.17	1.12 (0.96, 1.31)	0.17	1.09 (0.91, 1.30)	0.40
Isoleucine	0.061 (0.021) mmol/L	1.98 (1.65, 2.37)	6.32×10^{-13}	2.15 (1.82, 2.54)	3.21×10^{-18}	1.79 (1.49, 2.16)	3.45×10^{-09}	1.75 (1.45, 2.12)	2.27×10^{-08}	1.76 (1.46, 2.13)	2.32×10^{-08}	1.64 (1.33, 2.03)	1.29×10^{-05}
Lactate	2.40 (0.77) mmol/L	1.45 (1.28, 1.65)	3.47×10^{-08}	1.43 (1.25, 1.63)	2.41×10^{-07}	1.54 (1.33, 1.78)	3.51×10^{-08}	1.47 (1.26, 1.72)	2.22×10^{-06}	1.49 (1.28, 1.74)	9.78×10^{-07}	1.60 (1.33, 1.91)	1.21×10^{-06}
Leucine	0.071 (0.021) mmol/L	2.06 (1.73, 2.46)	2.15×10^{-15}	2.19 (1.86, 2.57)	6.16×10^{-20}	1.85 (1.54, 2.21)	1.40×10^{-10}	1.81 (1.51, 2.18)	1.79×10^{-09}	1.82 (1.51, 2.20)	1.99×10^{-09}	1.65 (1.34, 2.03)	5.31×10^{-06}
Mean diameter for HDL particles	9.90 (0.22) nm	0.43 (0.36, 0.51)	5.72×10^{-18}	0.42 (0.35, 0.50)	9.14×10^{-19}	0.44 (0.36, 0.54)	3.49×10^{-14}	0.43 (0.35, 0.53)	1.11×10^{-13}	0.43 (0.35, 0.53)	1.11×10^{-13}	0.42 (0.33, 0.53)	2.24×10^{-12}
Mean diameter for LDL particles	24.00 (0.10) nm	0.84 (0.72, 0.98)	0.04	0.84 (0.72, 0.98)	0.03	0.91 (0.77, 1.07)	0.29	0.95 (0.80, 1.13)	0.58	0.95 (0.80, 1.12)	0.57	1.03 (0.86, 1.24)	0.75
Mean diameter for VLDL particles	37.00 (1.30) nm	1.97 (1.66, 2.34)	4.90×10^{-14}	2.07 (1.76, 2.44)	1.93×10^{-17}	1.79 (1.50, 2.13)	6.97×10^{-10}	1.73 (1.44, 2.08)	1.32×10^{-08}	1.74 (1.45, 2.08)	1.34×10^{-08}	1.68 (1.38, 2.04)	9.09×10^{-07}
Monounsaturated fatty acids; 16:1, 18:1	3.00 (0.73) mmol/L	1.73(1.48, 2.02)	1.57×10^{-11}	1.74 (1.50, 2.03)	2.03×10^{-12}	1.50 (1.27, 1.77)	3.59×10^{-06}	1.47 (1.25, 1.73)	9.70×10^{-06}	1.47 (1.25, 1.73)	9.16×10^{-06}	1.48 (1.23, 1.77)	5.82×10^{-05}
Omega-3 fatty acids	0.41 (0.15) mmol/L	1.27 (1.09, 1.48)	2.50×10^{-03}	1.25 (1.07, 1.46)	0.01	1.07 (0.90, 1.27)	0.5	1.05 (0.88, 1.25)	0.58	1.05 (0.88, 1.25)	0.59	0.98 (0.81, 1.19)	0.87
Omega-6 fatty acids	3.20 (0.59) mmol/L	1.88 (1.60, 2.20)	5.03×10^{-14}	1.85 (1.57, 2.18)	5.16×10^{-13}	1.72 (1.44, 2.05)	6.25×10^{-09}	1.71 (1.43, 2.05)	1.81×10^{-08}	1.71 (1.43, 2.05)	1.89×10^{-08}	1.60 (1.32, 1.93)	3.23×10^{-06}
Phenylalanine	0.061 (0.0094) mmol/L	1.49 (1.30, 1.71)	2.36×10^{-08}	1.53 (1.33, 1.76)	5.22×10^{-09}	1.31 (1.10, 1.56)	4.10×10^{-03}	1.36 (1.15, 1.61)	5.10×10^{-04}	1.37 (1.16, 1.62)	4.20×10^{-04}	1.29 (1.05, 1.58)	0.03
Phosphatidylcholine and other cholines	1.60 (0.23) mmol/L	1.50 (1.30, 1.74)	1.17×10^{-07}	1.48 (1.27, 1.72)	7.18×10^{-07}	1.45 (1.23, 1.70)	1.78×10^{-05}	1.44 (1.22, 1.70)	3.46×10^{-05}	1.44 (1.22, 1.70)	3.47×10^{-05}	1.30 (1.08, 1.57)	8.30×10^{-03}
PL in chylomicrons and extremely large VLDL	0.0037 (0.003) mmol/L	2.03 (1.71, 2.41)	6.05×10^{-15}	2.16 (1.83, 2.54)	2.67×10^{-19}	1.85 (1.55, 2.20)	6.58×10^{-11}	1.79 (1.49, 2.14)	1.22×10^{-09}	1.79 (1.49, 2.14)	1.21×10^{-09}	1.65 (1.35, 2.02)	3.41×10^{-06}
PL in IDL	0.24 (0.053) mmol/L	0.87 (0.74, 1.01)	0.08	0.86 (0.73, 1.01)	0.07	0.88 (0.74, 1.06)	0.22	0.86 (0.72, 1.03)	0.13	0.86 (0.72, 1.03)	0.13	0.89 (0.74, 1.08)	0.29
PL in large HDL	0.32 (0.10) mmol/L	0.47 (0.39, 0.57)	6.35×10^{-14}	0.46 (0.38, 0.56)	1.86×10^{-14}	0.49 (0.40, 0.60)	1.63×10^{-11}	0.48 (0.39, 0.59)	6.54×10^{-11}	0.48 (0.39, 0.59)	6.98×10^{-11}	0.44 (0.35, 0.56)	8.99×10^{-11}
PL in large LDL	0.26 (0.052) mmol/L	0.98 (0.85, 1.14)	0.81	0.97 (0.84, 1.13)	0.74	0.97 (0.81, 1.15)	0.72	0.93 (0.78, 1.11)	0.45	0.93 (0.78, 1.11)	0.46	0.96 (0.80, 1.15)	0.71
PL in large VLDL	0.052 (0.035) mmol/L	1.97 (1.68, 2.32)	1.21×10^{-15}	2.06 (1.76, 2.40)	4.35×10^{-19}	1.76 (1.49, 2.08)	2.56×10^{-10}	1.71 (1.44, 2.03)	2.67×10^{-09}	1.71 (1.45, 2.03)	2.74×10^{-09}	1.66 (1.37, 2.00)	8.55×10^{-07}
PL in medium HDL	0.35 (0.056) mmol/L	1.01 (0.88, 1.16)	0.90	0.98 (0.85, 1.13)	0.78	0.91 (0.78, 1.06)	0.28	0.89 (0.76, 1.05)	0.19	0.89 (0.76, 1.05)	0.19	0.80 (0.67, 0.95)	0.02
PL in medium LDL	0.16 (0.03) mmol/L	1.32 (1.15, 1.52)	1.20×10^{-04}	1.30 (1.13, 1.50)	4.30×10^{-04}	1.25 (1.07, 1.47)	7.70×10^{-03}	1.21 (1.03, 1.42)	0.03	1.21 (1.03, 1.42)	0.03	1.20 (1.01, 1.42)	0.05
PL in medium VLDL	0.12 (0.056) mmol/L	2.01 (1.71, 2.36)	1.80×10^{-16}	2.07 (1.77, 2.42)	4.52×10^{-19}	1.76 (1.49, 2.08)	2.99×10^{-10}	1.72 (1.45, 2.04)	2.61×10^{-09}	1.72 (1.45, 2.04)	2.38×10^{-09}	1.73 (1.43, 2.09)	5.46×10^{-08}
PL in small HDL	0.56 (0.067) mmol/L	1.61 (1.39, 1.86)	3.25×10^{-10}	1.60 (1.38, 1.85)	5.24×10^{-10}	1.38 (1.18, 1.62)	1.50×10^{-04}	1.32 (1.12, 1.56)	1.40×10^{-03}	1.32 (1.12, 1.56)	1.40×10^{-03}	1.21 (1.02, 1.45)	0.05
PL in small LDL	0.12 (0.02) mmol/L	1.28 (1.12, 1.46)	5.40×10^{-04}	1.26 (1.10, 1.44)	1.60×10^{-03}	1.20 (1.02, 1.39)	0.03	1.15 (0.98, 1.34)	0.12	1.15 (0.98, 1.34)	0.11	1.12 (0.95, 1.32)	0.23
PL in small VLDL	0.13 (0.038) mmol/L	1.73 (1.47, 2.05)	1.44×10^{-10}	1.75 (1.50, 2.05)	7.19×10^{-12}	1.55 (1.31, 1.83)	6.59×10^{-07}	1.54 (1.30, 1.81)	8.72×10^{-07}	1.54 (1.30, 1.81)	8.16×10^{-07}	1.59 (1.32, 1.91)	2.39×10^{-06}
PL in very large HDL	0.21 (0.086) mmol/L	0.41 (0.34, 0.50)	1.78×10^{-17}	0.40 (0.33, 0.49)	6.18×10^{-18}	0.44 (0.35, 0.54)	4.34×10^{-13}	0.43 (0.34, 0.54)	1.53×10^{-12}	0.43 (0.34, 0.54)	1.71×10^{-12}	0.43 (0.34, 0.55)	3.66×10^{-11}
PL in very large VLDL	0.012 (0.01) mmol/L	1.91 (1.62, 2.26)	1.27×10^{-13}	2.00 (1.71, 2.35)	5.60×10^{-17}	1.70 (1.43, 2.03)	6.33×10^{-09}	1.65 (1.39, 1.97)	6.19×10^{-08}	1.66 (1.39, 1.97)	6.10×10^{-08}	1.54 (1.26, 1.89)	5.57×10^{-05}
PL in very small VLDL	0.12 (0.03) mmol/L	0.99 (0.85, 1.15)	0.89	0.98 (0.84, 1.14)	0.78	0.96 (0.81, 1.14)	0.69	0.93 (0.78, 1.11)	0.46	0.93 (0.78, 1.11)	0.46	0.98 (0.81, 1.18)	0.83

Metabolic biomarker	Mean (SD)	Model A		Model B		Model C1		Model C2		Model C3		Model D	
		HR* (95% CI)	*p*-value†	HR* (95% CI)	*p*-value†	HR* (95% CI)	*p*-value†	HR* (95% CI)	*p*-value†	HR* (95% CI)	*p*-value†	HR* (95% CI)	*p*-value†
PL:TL in chylomicrons and extremely large VLDL	10.00 (2.30) %	1.82 (1.54, 2.15)	6.16×10^{-12}	1.82 (1.54, 2.15)	9.60×10^{-12}	1.53 (1.29, 1.82)	3.27×10^{-06}	1.47 (1.23, 1.76)	6.53×10^{-05}	1.47 (1.23, 1.76)	6.69×10^{-05}	1.37 (1.12, 1.66)	3.60×10^{-03}
PL:TL in large VLDL	28.00 (1.10) %	2.24 (1.81, 2.79)	8.09×10^{-13}	2.22 (1.78, 2.76)	2.03×10^{-12}	2.07 (1.61, 2.65)	3.18×10^{-08}	1.96 (1.53, 2.51)	2.91×10^{-07}	1.97 (1.53, 2.52)	2.81×10^{-07}	1.85 (1.41, 2.43)	2.64×10^{-05}
PL:TL in medium HDL	50.00 (2.90) %	1.16 (1.03, 1.31)	0.02	1.16 (1.03, 1.30)	0.02	1.14 (1.00, 1.30)	0.08	1.14 (1.00, 1.31)	0.07	1.14 (1.00, 1.31)	0.07	1.26 (1.09, 1.45)	3.20×10^{-03}
PL:TL in medium LDL	27.00 (1.60) %	1.36 (1.17, 1.57)	8.17×10^{-05}	1.36 (1.17, 1.58)	1.40×10^{-04}	1.28 (1.08, 1.52)	7.30×10^{-03}	1.30 (1.09, 1.53)	4.50×10^{-03}	1.30 (1.09, 1.53)	4.60×10^{-03}	1.24 (1.04, 1.48)	0.02
PL:TL in medium VLDL	17.00 (0.79) %	0.76 (0.66, 0.88)	2.60×10^{-04}	0.75 (0.65, 0.87)	$2.00x10^{-04}$	0.90 (0.77, 1.06)	0.27	0.92 (0.78, 1.09)	0.36	0.92 (0.78, 1.09)	0.37	0.86 (0.72, 1.04)	0.16
PL:TL in small HDL	46.00 (1.70) %	1.42 (1.20, 1.68)	8.43×10^{-05}	1.43 (1.20, 1.70)	7.67×10^{-05}	1.28 (1.05, 1.54)	0.02	1.28 (1.06, 1.54)	0.02	1.28 (1.06, 1.54)	0.02	1.20 (0.98, 1.47)	0.10
PL:TL in small LDL	29.00 (3.70) %	1.29 (1.12, 1.49)	5.80×10^{-04}	1.29 (1.11, 1.49)	1.40×10^{-03}	1.22 (1.03, 1.44)	0.03	1.24 (1.05, 1.46)	0.02	1.23 (1.05, 1.46)	0.02	1.18 (1.00, 1.39)	0.08
PL:TL in small VLDL	20.00 (0.44) %	0.78 (0.68, 0.89)	2.70×10^{-04}	0.77 (0.68, 0.88)	2.60×10^{-04}	0.89 (0.77, 1.04)	0.18	0.92 (0.79, 1.08)	0.34	0.92 (0.79, 1.08)	0.35	0.87 (0.73, 1.03)	0.15
PL:TL in very large HDL	55.00 (3.60) %	0.72 (0.59, 0.86)	7.40×10^{-04}	0.70 (0.58, 0.85)	4.20×10^{-04}	0.82 (0.67, 1.00)	0.07	0.86 (0.70, 1.05)	0.17	0.86 (0.70, 1.05)	0.17	0.84 (0.67, 1.04)	0.16
PL:TL in very large VLDL	33.00 (4.40) %	1.76 (1.48, 2.09)	5.24×10^{-10}	1.73 (1.45, 2.06)	2.13×10^{-09}	1.51 (1.25, 1.83)	5.85×10^{-05}	1.45 (1.20, 1.75)	2.60×10^{-04}	1.45 (1.20, 1.76)	2.60×10^{-04}	1.28 (1.06, 1.56)	0.02
PL:TL in very small VLDL	24.00 (1.30) %	0.85 (0.74, 0.99)	0.04	0.85 (0.74, 0.98)	0.03	0.93 (0.80, 1.08)	0.36	0.94 (0.80, 1.09)	0.43	0.94 (0.80, 1.09)	0.44	0.89 (0.75, 1.04)	0.19
PL:TL in IDL	51.00 (6.50) %	0.82 (0.72, 0.94)	6.20×10^{-03}	0.83 (0.72, 0.95)	8.80×10^{-03}	0.93 (0.81, 1.07)	0.33	0.96 (0.84, 1.10)	0.57	0.96 (0.84, 1.10)	0.57	0.93 (0.78, 1.11)	0.47
PL:TL in large HDL	14.00 (2.60) %	1.53 (1.33, 1.76)	6.56×10^{-09}	1.53 (1.33, 1.77)	6.83×10^{-09}	1.36 (1.16, 1.59)	2.50×10^{-04}	1.29 (1.09, 1.52)	5.30×10^{-03}	1.29 (1.09, 1.52)	4.90×10^{-03}	1.31 (1.09, 1.58)	8.30×10^{-03}
PL:TL in large LDL	29.00 (2.50) %	1.30 (1.10, 1.54)	3.50×10^{-03}	1.30 (1.09, 1.55)	5.40×10^{-03}	1.24 (1.02, 1.52)	0.05	1.29 (1.05, 1.57)	0.02	1.28 (1.05, 1.57)	0.02	1.22 (1.00, 1.50)	0.08
Polyunsaturated fatty acids	3.60 (0.70) mmol/L	1.81 (1.55, 2.13)	9.92×10^{-13}	1.77 (1.50, 2.09)	2.27×10^{-11}	1.62 (1.36, 1.93)	2.82×10^{-07}	1.61 (1.35, 1.93)	4.69×10^{-07}	1.61 (1.35, 1.93)	4.79×10^{-07}	1.49 (1.24, 1.80)	6.72×10^{-05}
18:2 linoleic acid to TFA ratio	26.00 (2.80) %	1.04 (0.92, 1.17)	0.57	1.05 (0.93, 1.20)	0.44	1.21 (1.05, 1.41)	0.02	1.23 (1.05, 1.44)	0.01	1.23 (1.06, 1.44)	0.01	1.23 (1.04, 1.45)	0.03
22:6 docosahexaenoic acid to TFA ratio	1.20 (0.33) %	0.52 (0.46, 0.60)	2.09×10^{-18}	0.50 (0.44, 0.58)	3.52×10^{-19}	0.46 (0.39, 0.55)	5.47×10^{-16}	0.46 (0.38, 0.55)	1.53×10^{-14}	0.46 (0.38, 0.55)	1.48×10^{-14}	0.46 (0.37, 0.58)	1.09×10^{-10}
Apolipoprotein B:Apolipoprotein A-I	0.56 (0.11)	1.99 (1.67, 2.38)	1.04×10^{-13}	2.03 (1.69, 2.43)	8.51×10^{-14}	1.82 (1.51, 2.21)	2.80×10^{-09}	1.79 (1.48, 2.17)	8.68×10^{-09}	1.79 (1.48, 2.17)	8.92×10^{-09}	1.95 (1.58, 2.40)	2.29×10^{-09}
Monounsaturated fatty acids to TFA ratio	28.00 (2.10) %	1.54 (1.33, 1.79)	2.34×10^{-08}	1.56 (1.34, 1.81)	1.97×10^{-08}	1.34 (1.13, 1.58)	1.10×10^{-03}	1.30 (1.09, 1.54)	4.70×10^{-03}	1.30 (1.09, 1.54)	4.7×10^{-03}	1.38 (1.15, 1.66)	1.20×10^{-03}
Omega-3 fatty acids to TFA ratio	3.90 (0.82) %	0.84 (0.73, 0.96)	0.02	0.82 (0.71, 0.95)	0.01	0.73 (0.61, 0.87)	7.70×10^{-04}	0.72 (0.60, 0.87)	1.20×10^{-03}	0.72 (0.60, 0.87)	1.10×10^{-03}	0.67 (0.53, 0.85)	1.50×10^{-03}
Omega-6 fatty acids to TFA ratio	30.00 (2.70) %	0.97 (0.86, 1.09)	0.66	0.98 (0.87, 1.11)	0.78	1.14 (0.99, 1.31)	0.09	1.17 (1.01, 1.36)	0.05	1.17 (1.01, 1.36)	0.05	1.13 (0.96, 1.32)	0.19
Polyunsaturated fatty acids to TFA ratio	34.00 (2.60) %	0.93 (0.82, 1.05)	0.24	0.93 (0.82, 1.06)	0.30	1.07 (0.92, 1.23)	0.43	1.10 (0.95, 1.28)	0.24	1.10 (0.95, 1.28)	0.24	1.03 (0.88, 1.21)	0.74
Saturated fatty acids to TFA ratio	37.00 (1.60) %	0.61 (0.54, 0.70)	2.93×10^{-12}	0.60 (0.53, 0.69)	2.00×10^{-12}	0.62 (0.53, 0.73)	2.00×10^{-08}	0.62 (0.53, 0.73)	2.72×10^{-08}	0.62 (0.53, 0.73)	2.42×10^{-08}	0.61 (0.51, 0.73)	3.46×10^{-07}
TG:phosphoglycerides	0.68 (0.25)	1.94 (1.63, 2.30)	2.75×10^{-13}	2.02 (1.71, 2.39)	1.49×10^{-15}	1.73 (1.45, 2.07)	6.13×10^{-09}	1.69 (1.41, 2.03)	3.99×10^{-08}	1.69 (1.41, 2.03)	3.77×10^{-08}	1.72 (1.42, 2.08)	9.94×10^{-08}
Remnant cholesterol (non-HDL, non-LDL -cholesterol)	1.20 (0.32) mmol/L	1.24 (1.09, 1.42)	2.40×10^{-03}	1.23 (1.07, 1.41)	5.40×10^{-03}	1.12 (0.96, 1.31)	0.19	1.08 (0.93, 1.26)	0.36	1.08 (0.93, 1.26)	0.36	1.12 (0.95, 1.34)	0.24
Saturated fatty acids	3.90 (0.79) mmol/L	1.46 (1.26, 1.69)	6.81×10^{-07}	1.47 (1.27, 1.70)	2.76×10^{-07}	1.29 (1.11, 1.50)	1.90×10^{-03}	1.26 (1.09, 1.47)	4.10×10^{-03}	1.26 (1.09, 1.47)	4.30×10^{-03}	1.23 (1.04, 1.47)	0.03
Serum TC	3.70 (0.73) mmol/L	0.81 (0.71, 0.94)	6.20×10^{-03}	0.80 (0.70, 0.93)	4.90×10^{-03}	0.78 (0.66, 0.93)	6.70×10^{-03}	0.75 (0.63, 0.89)	1.70×10^{-03}	0.75 (0.63, 0.89)	1.70×10^{-03}	0.76 (0.63, 0.91)	5.00×10^{-03}
Serum total triglycerides	1.30 (0.51) mmol/L	2.06 (1.74, 2.42)	1.44×10^{-16}	2.13 (1.82, 2.49)	6.47×10^{-20}	1.82 (1.54, 2.16)	3.46×10^{-11}	1.78 (1.50, 2.11)	4.12×10^{-10}	1.78 (1.50, 2.11)	3.54×10^{-10}	1.78 (1.47, 2.16)	2.06×10^{-08}
Sphingomyelins	0.34 (0.057) mmol/L	1.24 (1.09, 1.41)	1.70×10^{-03}	1.22 (1.07, 1.39)	4.50×10^{-03}	1.21 (1.05, 1.39)	0.01	1.22 (1.05, 1.41)	0.01	1.22 (1.05, 1.41)	0.01	1.14 (0.98, 1.34)	0.13
TC in chylomicrons and extremely large VLDL	0.0054 (0.004) mmol/L	1.92 (1.62, 2.27)	1.11×10^{-13}	2.00 (1.70, 2.35)	1.03×10^{-16}	1.71 (1.44, 2.04)	3.71×10^{-09}	1.67 (1.40, 1.98)	2.72×10^{-08}	1.67 (1.40, 1.99)	2.76×10^{-08}	1.60 (1.32, 1.95)	6.26×10^{-06}
TC in HDL	1.30 (0.24) mmol/L	0.49 (0.42, 0.59)	4.08×10^{-15}	0.48 (0.40, 0.57)	6.43×10^{-16}	0.49 (0.41, 0.60)	4.27×10^{-12}	0.48 (0.39, 0.58)	6.24×10^{-12}	0.48 (0.39, 0.58)	6.43×10^{-12}	0.44 (0.35, 0.55)	6.28×10^{-12}
TC in HDL2	0.80 (0.22) mmol/L	0.50 (0.42, 0.59)	7.82×10^{-15}	0.48 (0.41, 0.57)	1.24×10^{-15}	0.50 (0.41, 0.61)	1.24×10^{-11}	0.49 (0.40, 0.60)	2.35×10^{-11}	0.49 (0.40, 0.60)	2.50×10^{-11}	0.45 (0.36, 0.56)	2.51×10^{-11}
TC in HDL3	0.46 (0.029) mmol/L	0.54 (0.47, 0.62)	1.15×10^{-15}	0.53 (0.46, 0.62)	3.80×10^{-16}	0.51 (0.43, 0.61)	2.04×10^{-13}	0.48 (0.40, 0.57)	3.81×10^{-14}	0.48 (0.40, 0.57)	3.26×10^{-14}	0.43 (0.36, 0.52)	7.31×10^{-16}
TC in IDL	0.53 (0.14) mmol/L	0.82 (0.70, 0.95)	0.01	0.81 (0.70, 0.95)	0.01	0.81 (0.68, 0.97)	0.03	0.79 (0.66, 0.94)	0.01	0.79 (0.66, 0.94)	0.01	0.82 (0.68, 0.99)	0.05
TC in large HDL	0.30 (0.12) mmol/L	0.45 (0.37, 0.55)	7.82×10^{-15}	0.44 (0.36, 0.54)	3.75×10^{-15}	0.48 (0.39, 0.59)	2.80×10^{-11}	0.48 (0.39, 0.60)	1.56×10^{-10}	0.48 (0.39, 0.60)	1.70×10^{-10}	0.45 (0.35, 0.57)	6.85×10^{-10}
TC in large LDL	0.66 (0.19) mmol/L	0.89 (0.76, 1.04)	0.16	0.89 (0.76, 1.04)	0.16	0.89 (0.75, 1.07)	0.27	0.86 (0.72, 1.03)	0.13	0.86 (0.72, 1.03)	0.14	0.90 (0.74, 1.08)	0.30
TC in large VLDL	0.067 (0.045) mmol/L	1.95 (1.66, 2.29)	3.21×10^{-15}	2.02 (1.73, 2.36)	5.25×10^{-18}	1.73 (1.46, 2.04)	9.03×10^{-10}	1.69 (1.43, 2.00)	5.41×10^{-09}	1.69 (1.43, 2.01)	5.48×10^{-09}	1.66 (1.38, 2.01)	5.54×10^{-07}
TC in LDL	1.30 (0.38) mmol/L	0.88 (0.76, 1.03)	0.14	0.88 (0.75, 1.03)	0.13	0.88 (0.74, 1.06)	0.22	0.85 (0.71, 1.02)	0.11	0.85 (0.71, 1.02)	0.11	0.87 (0.72, 1.05)	0.19
TC in medium HDL	0.37 (0.071) mmol/L	0.82 (0.72, 0.94)	6.80×10^{-03}	0.80 (0.70, 0.92)	2.50×10^{-03}	0.78 (0.67, 0.90)	1.90×10^{-03}	0.76 (0.66, 0.89)	1.10×10^{-03}	0.76 (0.66, 0.89)	1.10×10^{-03}	0.68 (0.57, 0.82)	9.01×10^{-05}
TC in medium LDL	0.37 (0.12) mmol/L	0.94 (0.81, 1.10)	0.49	0.94 (0.81, 1.09)	0.46	0.95 (0.80, 1.13)	0.58	0.91 (0.76, 1.09)	0.35	0.91 (0.76, 1.09)	0.36	0.94 (0.78, 1.12)	0.54
TC in medium VLDL	0.16 (0.075) mmol/L	1.88 (1.61, 2.20)	1.79×10^{-14}	1.91 (1.63, 2.23)	1.28×10^{-15}	1.63 (1.38, 1.93)	3.31×10^{-08}	1.60 (1.36, 1.90)	9.57×10^{-08}	1.61 (1.36, 1.90)	8.81×10^{-08}	1.65 (1.37, 1.98)	3.39×10^{-07}
TC in small HDL	0.41 (0.056) mmol/L	0.84 (0.72, 0.98)	0.03	0.83 (0.71, 0.97)	0.02	0.84 (0.71, 0.99)	0.06	0.81 (0.69, 0.96)	0.02	0.81 (0.69, 0.96)	0.03	0.79 (0.66, 0.95)	0.02
TC in small LDL	0.22 (0.073) mmol/L	0.89 (0.77, 1.04)	0.16	0.89 (0.76, 1.04)	0.16	0.90 (0.76, 1.07)	0.29	0.87 (0.72, 1.04)	0.15	0.87 (0.72, 1.04)	0.15	0.90 (0.75, 1.07)	0.29
TC in small VLDL	0.19 (0.064) mmol/L	1.19 (1.04, 1.35)	0.01	1.18 (1.03, 1.35)	0.02	1.08 (0.93, 1.25)	0.37	1.04 (0.90, 1.21)	0.60	1.04 (0.90, 1.21)	0.61	1.11 (0.94, 1.31)	0.28
TC in very large HDL	0.19 (0.079) mmol/L	0.47 (0.40, 0.56)	5.72×10^{-18}	0.47 (0.40, 0.56)	1.93×10^{-17}	0.48 (0.40, 0.58)	3.31×10^{-13}	0.47 (0.39, 0.57)	1.73×10^{-13}	0.47 (0.39, 0.57)	1.43×10^{-13}	0.47 (0.38, 0.58)	7.53×10^{-12}
TC in very large VLDL	0.016 (0.012) mmol/L	1.84 (1.56, 2.17)	2.71×10^{-12}	1.91 (1.63, 2.25)	6.25×10^{-15}	1.64 (1.38, 1.95)	6.30×10^{-08}	1.60 (1.34, 1.90)	3.76×10^{-07}	1.60 (1.34, 1.91)	3.71×10^{-07}	1.52 (1.24, 1.85)	9.01×10^{-05}
TC in very small VLDL	0.19 (0.054) mmol/L	0.73 (0.64, 0.84)	2.58×10^{-05}	0.73 (0.63, 0.84)	2.01×10^{-05}	0.70 (0.59, 0.83)	6.64×10^{-05}	0.67 (0.56, 0.79)	1.24×10^{-05}	0.67 (0.56, 0.79)	1.20×10^{-05}	0.71 (0.59, 0.85)	$5.00x10^{-04}$
TC in VLDL	0.62 (0.22) mmol/L	1.49 (1.29, 1.73)	1.01×10^{-07}	1.49 (1.29, 1.72)	1.21×10^{-07}	1.30 (1.11, 1.52)	1.70×10^{-03}	1.27 (1.09, 1.48)	4.10×10^{-03}	1.27 (1.09, 1.48)	4.30×10^{-3}	1.32 (1.11, 1.57)	3.10×10^{-03}
TC:TL in chylomicrons and extremely large VLDL	16.00 (4.50) %	0.92 (0.82, 1.03)	0.17	0.92 (0.82, 1.04)	0.22	0.92 (0.80, 1.07)	0.33	0.94 (0.80, 1.09)	0.43	0.94 (0.80, 1.09)	0.43	0.97 (0.82, 1.15)	0.78
TC:TL in IDL	61.00 (2.90) %	0.68 (0.57, 0.81)	3.15×10^{-05}	0.67 (0.56, 0.80)	1.37×10^{-05}	0.69 (0.58, 0.83)	1.40×10^{-04}	0.67 (0.56, 0.81)	6.19×10^{-05}	0.67 (0.56, 0.81)	6.02×10^{-05}	0.65 (0.54, 0.77)	2.73×10^{-06}
TC:TL in large HDL	46.00 (3.40) %	0.53 (0.45, 0.62)	6.83×10^{-14}	0.51 (0.43, 0.60)	4.74×10^{-15}	0.54 (0.46, 0.64)	3.13×10^{-11}	0.56 (0.47, 0.67)	1.05×10^{-09}	0.56 (0.47, 0.67)	8.18×10^{-10}	0.55 (0.46, 0.67)	2.29×10^{-09}
TC:TL in large LDL	65.00 (3.10) %	0.65 (0.55, 0.77)	1.21×10^{-06}	0.65 (0.54, 0.77)	2.35×10^{-06}	0.69 (0.57, 0.84)	4.10×10^{-04}	0.67 (0.55, 0.81)	1.20×10^{-04}	0.67 (0.55, 0.81)	1.10×10^{-04}	0.70 (0.58, 0.85)	6.90×10^{-04}
TC:TL in large VLDL	22.00 (2.20) %	1.15 (1.00, 1.33)	0.07	1.15 (0.99, 1.33)	0.09	1.18 (0.99, 1.42)	0.08	1.20 (1.00, 1.43)	0.07	1.20 (1.00, 1.43)	0.07	1.26 (1.02, 1.55)	0.04
TC:TL in medium HDL	48.00 (2.90) %	0.67 (0.58, 0.77)	1.32×10^{-07}	0.67 (0.58, 0.77)	3.15×10^{-08}	0.70 (0.60, 0.83)	3.77×10^{-05}	0.71 (0.60, 0.83)	6.76×10^{-05}	0.71 (0.60, 0.83)	6.80×10^{-05}	0.60 (0.52, 0.68)	1.56×10^{-12}
TC:TL in medium LDL	63.00 (5.30) %	0.73 (0.63, 0.85)	4.32×10^{-05}	0.73 (0.63, 0.85)	7.56×10^{-05}	0.77 (0.65, 0.91)	3.90×10^{-03}	0.76 (0.64, 0.90)	2.00×10^{-03}	0.76 (0.64, 0.90)	2.00×10^{-03}	0.79 (0.67, 0.94)	0.01
TC:TL in medium VLDL	26.00 (2.80) %	0.78 (0.69, 0.89)	3.30×10^{-04}	0.78 (0.69, 0.90)	5.40×10^{-04}	0.83 (0.72, 0.96)	0.02	0.84 (0.72, 0.98)	0.03	0.84 (0.72, 0.98)	0.03	0.92 (0.77, 1.10)	0.43
TC:TL in small HDL	40.00 (4.20) %	0.60 (0.51, 0.72)	5.19×10^{-08}	0.58 (0.49, 0.69)	1.82×10^{-09}	0.65 (0.54, 0.79)	2.02×10^{-05}	0.66 (0.54, 0.79)	2.34×10^{-05}	0.66 (0.54, 0.79)	2.33×10^{-05}	0.70 (0.57, 0.85)	8.40×10^{-04}

Metabolic biomarker	Mean (SD)	Model A		Model B		Model C1		Model C2		Model C3		Model D	
		**HR* (95% CI)**	***p*-value†**	**HR* (95% CI)**	***p*-value†**	**HR* (95% CI)**	***p*-value†**	**HR* (95% CI)**	***p*-value†**	**HR* (95% CI)**	***p*-value†**	**HR* (95% CI)**	***p*-value†**
TC:TL in small LDL	59.00 (6.20) %	0.72 (0.62, 0.83)	$1.38\times10^{-0}5$	0.72 (0.62, 0.83)	1.95×10^{-05}	0.77 (0.65, 0.90)	2.40×10^{-03}	0.76 (0.64, 0.89)	1.50×10^{-03}	0.76 (0.64, 0.89)	1.60×10^{-03}	0.79 (0.67, 0.94)	0.01
TC:TL in small VLDL	33.00 (5.60) %	0.58 (0.50, 0.67)	7.86×10^{-12}	0.56 (0.48, 0.65)	1.37×10^{-12}	0.58 (0.48, 0.69)	6.87×10^{-09}	0.57 (0.47, 0.68)	6.74×10^{-09}	0.56 (0.47, 0.68)	5.82×10^{-09}	0.57 (0.47, 0.69)	2.93×10^{-08}
TC:TL in very large HDL	45.00 (5.60) %	0.91 (0.77, 1.07)	0.28	0.91 (0.77, 1.08)	0.31	0.84 (0.71, 1.00)	0.07	0.80 (0.67, 0.96)	0.02	0.80 (0.67, 0.96)	0.02	0.83 (0.68, 1.01)	0.09
TC:TL in very large VLDL	20.00 (3.80) %	0.84 (0.73, 0.96)	0.02	0.83 (0.72, 0.96)	0.02	0.90 (0.76, 1.06)	0.26	0.93 (0.79, 1.11)	0.46	0.93 (0.79, 1.11)	0.46	0.95 (0.81, 1.12)	0.61
TC:TL in very small VLDL	46.00 (4.80) %	0.52 (0.41, 0.65)	1.53×10^{-08}	0.48 (0.40, 0.57)	8.19×10^{-16}	0.50 (0.41, 0.60)	1.08×10^{-11}	0.49 (0.40, 0.59)	6.16×10^{-12}	0.49 (0.40, 0.59)	4.24×10^{-12}	0.50 (0.41, 0.61)	9.51×10^{-11}
Total cholines	2.00 (0.26) mmol/L	1.09 (0.96, 1.25)	0.21	1.08 (0.94, 1.24)	0.31	1.05 (0.90, 1.23)	0.54	1.05 (0.90, 1.22)	0.59	1.05 (0.90, 1.22)	0.59	0.97 (0.81, 1.15)	0.74
Total fatty acids	10.00 (2.10) mmol/L	1.69 (1.44, 1.97)	1.96×10^{-10}	1.68 (1.44, 1.97)	1.02×10^{-10}	1.47 (1.25, 1.74)	1.06×10^{-05}	1.45 (1.23, 1.71)	1.84×10^{-05}	1.45 (1.23, 1.71)	1.82×10^{-05}	1.41 (1.18, 1.69)	4.10×10^{-04}
TL in chylomicrons and extremely large VLDL	0.032 (0.023) mmol/L	2.05 (1.72, 2.43)	2.73×10^{-15}	2.18 (1.85, 2.56)	1.26×10^{-19}	1.87 (1.56, 2.22)	3.13×10^{-11}	1.80 (1.51, 2.16)	5.99×10^{-10}	1.81 (1.51, 2.16)	6.09×10^{-10}	1.68 (1.37, 2.05)	1.34×10^{-06}
TL in IDL	0.87 (0.21) mmol/L	0.90 (0.77, 1.04)	0.18	0.89 (0.76, 1.04)	0.16	0.89 (0.75, 1.06)	0.26	0.87 (0.73, 1.03)	0.14	0.87 (0.73, 1.03)	0.14	0.90 (0.75, 1.09)	0.33
TL in large HDL	0.65 (0.23) mmol/L	0.47 (0.39, 0.57)	5.13×10^{-14}	0.46 (0.38, 0.56)	2.21×10^{-14}	0.50 (0.41, 0.61)	5.38×10^{-11}	0.49 (0.40, 0.61)	2.69×10^{-10}	0.49 (0.40, 0.61)	2.86×10^{-10}	0.46 (0.36, 0.58)	6.85×10^{-10}
TL in large LDL	1.00 (0.25) mmol/L	0.95 (0.82, 1.11)	0.54	0.94 (0.81, 1.10)	0.49	0.95 (0.79, 1.13)	0.57	0.91 (0.76, 1.09)	0.35	0.91 (0.76, 1.09)	0.35	0.94 (0.79, 1.14)	0.60
TL in large VLDL	0.30 (0.19) mmol/L	1.97 (1.67, 2.31)	1.66×10^{-15}	2.05 (1.76, 2.39)	5.73×10^{-19}	1.75 (1.48, 2.07)	3.45×10^{-10}	1.70 (1.44, 2.02)	3.65×10^{-09}	1.71 (1.44, 2.02)	3.67×10^{-09}	1.65 (1.36, 1.99)	1.07×10^{-06}
TL in medium HDL	0.77 (0.13) mmol/L	0.98 (0.86, 1.12)	0.77	0.95 (0.83, 1.09)	0.50	0.89 (0.76, 1.03)	0.16	0.87 (0.75, 1.02)	0.10	0.87 (0.75, 1.02)	0.10	0.78 (0.66, 0.93)	0.01
TL in medium LDL	0.57 (0.15) mmol/L	1.05 (0.91, 1.21)	0.54	1.04 (0.90, 1.21)	0.61	1.04 (0.88, 1.23)	0.68	1.00 (0.84, 1.19)	0.99	1.00 (0.84, 1.19)	1.00	1.02 (0.85, 1.21)	0.87
TL in medium VLDL	0.60 (0.29) mmol/L	2.00 (1.70, 2.34)	2.40×10^{-16}	2.06 (1.76, 2.40)	5.17×10^{-19}	1.74 (1.47, 2.06)	4.20×10^{-10}	1.70 (1.44, 2.02)	3.82×10^{-09}	1.71 (1.44, 2.02)	3.67×10^{-09}	1.71 (1.42, 2.07)	7.97×10^{-08}
TL in small HDL	1.00 (0.094) mmol/L	1.40 (1.23, 1.59)	4.06×10^{-07}	1.38 (1.22, 1.57)	1.29×10^{-06}	1.23 (1.07, 1.41)	4.40×10^{-03}	1.18 (1.02, 1.36)	0.03	1.18 (1.02, 1.36)	0.01	1.08 (0.92, 1.27)	0.40
TL in small LDL	0.37 (0.094) mmol/L	1.03 (0.89, 1.19)	0.69	1.02 (0.88, 1.18)	0.78	1.01 (0.85, 1.20)	0.91	0.97 (0.81, 1.15)	0.73	0.97 (0.81, 1.15)	0.74	0.98 (0.83, 1.17)	0.87
TL in small VLDL	0.56 (0.18) mmol/L	1.74 (1.48, 2.05)	4.45×10^{-11}	1.75 (1.50, 2.05)	4.58×10^{-12}	1.53 (1.30, 1.81)	1.33×10^{-06}	1.51 (1.28, 1.78)	2.34×10^{-06}	1.51 (1.28, 1.78)	2.24×10^{-06}	1.56 (1.30, 1.88)	4.00×10^{-06}
TL in very large HDL	0.41 (0.16) mmol/L	0.44 (0.37, 0.53)	1.40×10^{-17}	0.44 (0.37, 0.53)	1.95×10^{-17}	0.46 (0.38, 0.56)	5.77×10^{-13}	0.45 (0.37, 0.55)	8.25×10^{-13}	0.45 (0.37, 0.55)	7.38×10^{-13}	0.45 (0.36, 0.56)	2.75×10^{-11}
TL in very large VLDL	0.079 (0.058) mmol/L	1.91 (1.62, 2.26)	1.15×10^{-13}	2.01 (1.72, 2.36)	3.07×10^{-17}	1.71 (1.44, 2.04)	4.27×10^{-09}	1.66 (1.39, 1.98)	4.45×10^{-08}	1.67 (1.40, 1.99)	4.39×10^{-08}	1.56 (1.27, 1.90)	4.07×10^{-05}
TL in very small VLDL	0.42 (0.098) mmol/L	1.04 (0.91, 1.20)	0.56	1.03 (0.90, 1.19)	0.66	0.98 (0.83, 1.14)	0.78	0.94 (0.80, 1.10)	0.47	0.94 (0.80, 1.10)	0.47	1.00 (0.83, 1.20)	0.99
Total phosphoglycerides	1.80 (0.27) mmol/L	1.08 (0.95, 1.23)	0.26	1.06 (0.93, 1.21)	0.41	1.00 (0.86, 1.16)	0.99	0.98 (0.84, 1.14)	0.80	0.98 (0.84, 1.14)	0.80	0.92 (0.77, 1.09)	0.40
TG in chylomicrons and extremely large VLDL	0.023 (0.016) mmol/L	2.07 (1.74, 2.46)	1.58×10^{-15}	2.22 (1.88, 2.61)	6.16×10^{-20}	1.90 (1.59, 2.27)	1.29×10^{-11}	1.84 (1.53, 2.20)	2.65×10^{-10}	1.84 (1.54, 2.20)	2.63×10^{-10}	1.70 (1.39, 2.07)	9.09×10^{-07}
TG in HDL	0.13 (0.034) mmol/L	2.12 (1.79, 2.52)	1.48×10^{-16}	2.14 (1.82, 2.53)	9.14×10^{-19}	1.88 (1.57, 2.25)	2.93×10^{-11}	1.86 (1.56, 2.23)	1.00×10^{-10}	1.87 (1.56, 2.23)	8.47×10^{-11}	1.89 (1.53, 2.32)	9.99×10^{-09}
TG in IDL	0.095 (0.023) mmol/L	1.53 (1.30, 1.79)	5.78×10^{-07}	1.54 (1.31, 1.81)	2.32×10^{-07}	1.45 (1.22, 1.71)	3.08×10^{-05}	1.43 (1.21, 1.70)	6.53×10^{-05}	1.43 (1.21, 1.70)	6.65×10^{-05}	1.65 (1.40, 1.95)	1.02×10^{-08}
TG in large HDL	0.025 (0.011) mmol/L	1.17 (1.02, 1.35)	0.04	1.17 (1.01, 1.35)	0.04	1.21 (1.03, 1.43)	0.03	1.27 (1.08, 1.51)	7.40×10^{-03}	1.27 (1.08, 1.51)	7.80×10^{-03}	1.23 (1.00, 1.52)	0.01
TG in large LDL	0.078 (0.02) mmol/L	1.42 (1.22, 1.65)	1.20×10^{-05}	1.42 (1.22, 1.65)	6.82×10^{-06}	1.35 (1.15, 1.58)	4.60×10^{-04}	1.34 (1.14, 1.58)	8.70×10^{-04}	1.34 (1.14, 1.57)	8.80×10^{-04}	1.50 (1.27, 1.77)	4.58×10^{-06}
TG in large VLDL	0.18 (0.11) mmol/L	1.97 (1.67, 2.31)	1.81×10^{-15}	2.05 (1.76, 2.39)	4.57×10^{-19}	1.75 (1.48, 2.07)	3.45×10^{-10}	1.70 (1.44, 2.02)	4.04×10^{-09}	1.70 (1.44, 2.02)	4.10×10^{-09}	1.63 (1.35, 1.98)	1.58×10^{-06}
TG in LDL	0.14 (0.036) mmol/L	1.48 (1.26, 1.73)	1.72×10^{-06}	1.48 (1.27, 1.72)	8.28×10^{-07}	1.39 (1.19, 1.64)	1.20×10^{-04}	1.38 (1.17, 1.63)	2.60×10^{-04}	1.38 (1.17, 1.63)	2.60×10^{-04}	1.53 (1.30, 1.81)	1.76×10^{-06}
TG in medium HDL	0.046 (0.012) mmol/L	2.27 (1.89, 2.73)	1.78×10^{-17}	2.31 (1.95, 2.74)	4.92×10^{-20}	2.02 (1.69, 2.42)	4.76×10^{-13}	2.01 (1.68, 2.41)	9.52×10^{-13}	2.02 (1.68, 2.43)	7.38×10^{-13}	2.04 (1.67, 2.49)	3.66×10^{-11}
TG in medium LDL	0.039 (0.0097) mmol/L	1.44 (1.24, 1.68)	5.71×10^{-06}	1.44 (1.24, 1.68)	3.16×10^{-06}	1.37 (1.17, 1.61)	2.10×10^{-04}	1.36 (1.15, 1.60)	4.60×10^{-04}	1.36 (1.15, 1.60)	4.60×10^{-04}	1.52 (1.29, 1.78)	1.51×10^{-06}
TG in medium VLDL	0.32 (0.16) mmol/L	2.02(1.72, 2.38)	1.94×10^{-16}	2.10 (1.80, 2.45)	1.03×10^{-19}	1.78 (1.50, 2.11)	1.28×10^{-10}	1.73 (1.46, 2.06)	1.71×10^{-09}	1.74 (1.46, 2.06)	1.61×10^{-09}	1.72 (1.43, 2.08)	7.97×10^{-08}
TG in small HDL	0.05 (0.013) mmol/L	2.59 (2.14, 3.12)	2.71×10^{-21}	2.71 (2.26, 3.24)	1.78×10^{-25}	2.40 (1.99, 2.90)	1.44×10^{-17}	2.35 (1.94, 2.85)	5.64×10^{-16}	2.36 (1.94, 2.86)	5.79×10^{-16}	2.50 (2.03, 3.09)	7.31×10^{-16}
TG in small LDL	0.026 (0.0074) mmol/L	1.73 (1.47, 2.04)	1.45×10^{-10}	1.75 (1.49, 2.05)	2.26×10^{-11}	1.57 (1.32, 1.85)	4.61×10^{-07}	1.54 (1.30, 1.82)	1.48×10^{-06}	1.54 (1.30, 1.82)	1.50×10^{-06}	1.61 (1.34, 1.93)	1.21×10^{-06}
TG in small VLDL	0.24 (0.092) mmol/L	2.13 (1.80, 2.53)	3.93×10^{-17}	2.19 (1.86, 2.58)	1.38×10^{-19}	1.88 (1.57, 2.24)	2.98×10^{-11}	1.85 (1.55, 2.21)	1.34×10^{-10}	1.86 (1.55, 2.22)	1.05×10^{-10}	1.88 (1.55, 2.28)	1.63×10^{-09}
TG in very large HDL	0.015 (0.0068) mmol/L	1.29 (1.11, 1.50)	1.70×10^{-03}	1.28 (1.10, 1.50)	2.00×10^{-03}	1.17 (0.99, 1.38)	0.09	1.17 (0.99, 1.38)	0.08	1.17 (0.99, 1.38)	0.09	1.15 (0.95, 1.41)	0.20
TG in very large VLDL	0.051 (0.037) mmol/L	1.94 (1.64, 2.29)	5.50×10^{-14}	2.04 (1.74, 2.39)	8.95×10^{-18}	1.74 (1.46, 2.07)	2.06×10^{-09}	1.68 (1.41, 2.01)	2.72×10^{-08}	1.69 (1.41, 2.01)	2.65×10^{-08}	1.57 (1.28, 1.92)	3.45×10^{-05}
TG in very small VLDL	0.10 (0.028) mmol/L	1.82 (1.53, 2.16)	2.04×10^{-11}	1.86 (1.58, 2.19)	4.67×10^{-13}	1.67 (1.40, 1.98)	1.81×10^{-08}	1.65 (1.39, 1.97)	4.35×10^{-08}	1.65 (1.39, 1.97)	3.94×10^{-08}	1.78 (1.48, 2.15)	1.08×10^{-08}
TG in VLDL	0.90 (0.44) mmol/L	2.04 (1.73, 2.40)	1.51×10^{-16}	2.12 (1.82, 2.48)	6.47×10^{-20}	1.80 (1.52, 2.14)	5.99×10^{-11}	1.76 (1.48, 2.08)	8.53×10^{-10}	1.76 (1.48, 2.09)	7.72×10^{-10}	1.74 (1.44, 2.11)	5.46×10^{-08}
TG:TL in chylomicrons and extremely large VLDL	73.00 (5.40) %	0.89 (0.80, 1.00)	0.05	0.89 (0.79, 1.00)	0.05	0.92 (0.81, 1.06)	0.29	0.93 (0.80, 1.07)	0.35	0.93 (0.80, 1.07)	0.35	0.92 (0.78, 1.08)	0.34
TG:TL in IDL	11.00 (2.80) %	1.81 (1.52, 2.16)	1.44×10^{-10}	1.88 (1.58, 2.23)	4.02×10^{-12}	1.73 (1.43, 2.10)	4.80×10^{-08}	1.77 (1.46, 2.13)	1.49×10^{-08}	1.77 (1.47, 2.14)	1.10×10^{-08}	1.76 (1.44, 2.14)	1.11×10^{-07}
TG:TL in large HDL	4.10 (1.70) %	2.18 (1.76, 2.71)	5.58×10^{-12}	2.24 (1.81, 2.76)	2.93×10^{-13}	2.07 (1.68, 2.56)	5.70×10^{-11}	2.07 (1.67, 2.55)	1.34×10^{-10}	2.07 (1.67, 2.56)	1.41×10^{-10}	2.12 (1.69, 2.65)	4.14×10^{-10}
TG:TL in large LDL	8.10 (2.00) %	1.62 (1.37, 1.92)	3.92×10^{-08}	1.63 (1.38, 1.93)	3.82×10^{-08}	1.52 (1.26, 1.82)	2.21×10^{-05}	1.55 (1.29, 1.86)	8.09×10^{-06}	1.55 (1.29, 1.86)	7.87×10^{-06}	1.52 (1.25, 1.85)	6.72×10^{-05}
TG:TL in large VLDL	60.00 (2.80) %	0.74 (0.63, 0.87)	2.80×10^{-04}	0.75 (0.64, 0.88)	5.40×10^{-04}	0.74 (0.61, 0.90)	4.00×10^{-03}	0.74 (0.61, 0.90)	4.70×10^{-03}	0.74 (0.61, 0.90)	4.70×10^{-03}	0.72 (0.58, 0.91)	8.30×10^{-03}
TG:TL in medium HDL	6.00 (1.70) %	2.02 (1.69, 2.41)	2.38×10^{-14}	2.07 (1.73, 2.46)	1.49×10^{-15}	1.88 (1.56, 2.27)	2.99×10^{-10}	1.87 (1.54, 2.27)	1.05×10^{-09}	1.88 (1.55, 2.28)	9.92×10^{-10}	2.22 (1.84, 2.68)	4.61×10^{-15}
TG:TL in medium LDL	7.10 (1.90) %	1.37 (1.19, 1.58)	2.74×10^{-05}	1.37 (1.18, 1.58)	4.68×10^{-05}	1.30 (1.11, 1.52)	2.00×10^{-03}	1.33 (1.13, 1.55)	8.70×10^{-04}	1.33 (1.13, 1.55)	8.80×10^{-04}	1.30 (1.10, 1.55)	4.30×10^{-03}
TG:TL in medium VLDL	54.00 (3.00) %	1.31(1.15, 1.49)	8.74×10^{-05}	1.32 (1.15, 1.51)	1.40×10^{-04}	1.22 (1.05, 1.41)	0.02	1.20 (1.03, 1.40)	0.03	1.20 (1.03, 1.40)	0.03	1.11 (0.93, 1.34)	0.30
TG:TL in small HDL	4.90 (1.20) %	2.11 (1.76, 2.53)	3.31×10^{-15}	2.18 (1.83, 2.60)	1.58×10^{-17}	1.96 (1.62, 2.35)	1.30×10^{-11}	1.94 (1.60, 2.34)	8.70×10^{-11}	1.94 (1.61, 2.35)	8.02×10^{-11}	2.17 (1.78, 2.65)	5.12×10^{-13}
TG:TL in small LDL	7.40 (2.20) %	1.54 (1.31, 1.80)	1.52×10^{-07}	1.58 (1.35, 1.84)	1.15×10^{-08}	1.45 (1.23, 1.71)	3.08×10^{-05}	1.45 (1.23, 1.72)	2.67×10^{-05}	1.45 (1.23, 1.72)	2.76×10^{-05}	1.40 (1.17, 1.67)	4.50×10^{-04}
TG:TL in small VLDL	43.00 (5.50) %	1.89 (1.59, 2.26)	3.71×10^{-12}	1.99 (1.68, 2.37)	1.13×10^{-14}	1.86 (1.54, 2.25)	4.91×10^{-10}	1.88 (1.55, 2.28)	1.02×10^{-09}	1.88 (1.55, 2.28)	8.18×10^{-10}	1.86 (1.52, 2.27)	1.27×10^{-08}
TG:TL in very large HDL	3.90 (2.20) %	2.17 (1.79, 2.63)	2.24×10^{-14}	2.34 (1.93, 2.83)	1.55×10^{-17}	2.28 (1.89, 2.76)	5.47×10^{-16}	2.26 (1.86, 2.75)	2.09×10^{-14}	2.26 (1.86, 2.75)	2.12×10^{-14}	2.21 (1.84, 2.66)	2.88×10^{-15}
TG:TL in very large VLDL	65.00 (4.90) %	0.92 (0.81, 1.05)	0.23	0.93 (0.81, 1.06)	0.30	0.92 (0.78, 1.07)	0.30	0.89 (0.76, 1.05)	0.20	0.89 (0.76, 1.05)	0.20	0.94 (0.80, 1.10)	0.48
TG:TL in very small VLDL	24.00 (5.50) %	1.90 (1.56, 2.31)	3.25×10^{-10}	2.01 (1.66, 2.42)	8.59×10^{-13}	1.87 (1.52, 2.30)	6.87×10^{-09}	1.90 (1.54, 2.33)	5.37×10^{-09}	1.90 (1.55, 2.34)	4.10×10^{-09}	1.88 (1.54, 2.31)	4.87×10^{-09}
Tyrosine	0.053 (0.013) mmol/L	1.49 (1.32, 1.69)	5.75×10^{-10}	1.49 (1.32, 1.68)	5.43×10^{-10}	1.25 (1.09, 1.45)	3.40×10^{-03}	1.21 (1.04, 1.40)	0.02	1.21 (1.04, 1.40)	0.02	0.97 (0.80, 1.17)	0.78
Valine	0.17 (0.041) mmol/L	2.25 (1.94, 2.62)	2.10×10^{-23}	2.39 (2.04, 2.79)	1.78×10^{-25}	2.00 (1.68, 2.38)	2.95×10^{-13}	2.01 (1.68, 2.40)	2.38×10^{-13}	2.05 (1.71, 2.45)	1.11×10^{-13}	1.91 (1.57, 2.32)	4.38×10^{-10}

Model A: adjusted for age, sex, study area, education, fasting time; Model B: additionally adjusted for smoking, alcohol, physical activity, dietary factors (meat, fish, fresh fruit, dairy), family history of diabetes; Model C1: additionally adjusted for BMI; Model C2: model B additionally adjusted for waist circumference; Model C3: model B additionally adjusted for BMI and waist circumference; Model D: Model C3 additionally adjusted for random plasma glucose.

*HR per 1 SD higher metabolic biomarker

†Adjusted for multiple testing using Benjamini-Hochberg correction (Journal of the Royal Statistical Society B 1995;57:289-300)

CE=cholesterol esters; FC=free cholesterol; PL=phospholipids; TC=total cholesterol; TFA=total fatty acids; TG=triglycerides; TL=total lipids

;

Supplementary Table S2. Chinese population type 2 diabetes risk prediction models

Study	No. of participants	No. of diabetes cases	Type 2 diabetes definition	Follow-up period	Variables included	AUC
Chin-Shan Community Cardiovascular Cohort Study*	2,960	548	FPG ≥7.0 mmol/L, oral hypoglycaemic med or insulin use	10 y	Age, FPG, BMI, TG, WBC, HDL-C	0.702
Chinese Diabetes Risk Score†	Derivation: 1,457<br>Validation: 394	Derivation: 304<br>Validation: 48	Self-reported diagnosis, use of hypoglycaemic meds, FPG ≥7.0 mmol/L, 2h post-load glucose ≥11.1 mmol/L	10 y	Age, HTN, history of elevated blood glucose, BMI, FPG, TG, HDL-C	0.722
Guangzhou Biobank Cohort‡	Derivation: 8,000<br>Validation: 8,043	836	Self-reported physician diagnosis, hypoglycemic meds or insulin, FPG ≥7.0 mmol/L	4.1 y	BMI, BP, antihypertensive medication use, HTN, HDL-C, TG, FPG	0.779
Kailuan§	Derivation: 49,325<br>Validation: 24,662	Derivation: 4,726<br>Validation: 2,327	Self-reported diabetes diagnosis, taking anti-diabetic meds, FPG ≥7.0 mmol/L	5 y	Age, sex, BMI, FH of diabetes, education, BP, HR, FPG, TG, lipid-lowering medication use	0.77

*Diabetologia 2009;52:443–450

†Diab Tech Ther 2011;13(5):501-507

‡Prev Med 2014;69:63–68

§Sci Rep 2016;6:26548

AUC=area under the curve; BMI=body mass index; BP=blood pressure; FH= family history; FPG=fasting plasma glucose; HDL-C=HDL-cholesterol; HR=heart rate; HTN=hypertension; TG=triglycerides; WBC=white blood cell count

Supplementary Figure S1. Correlation between directly-measured metabolic biomarkers within subcohort participants (n=789)

Adjusted for age, sex and study area

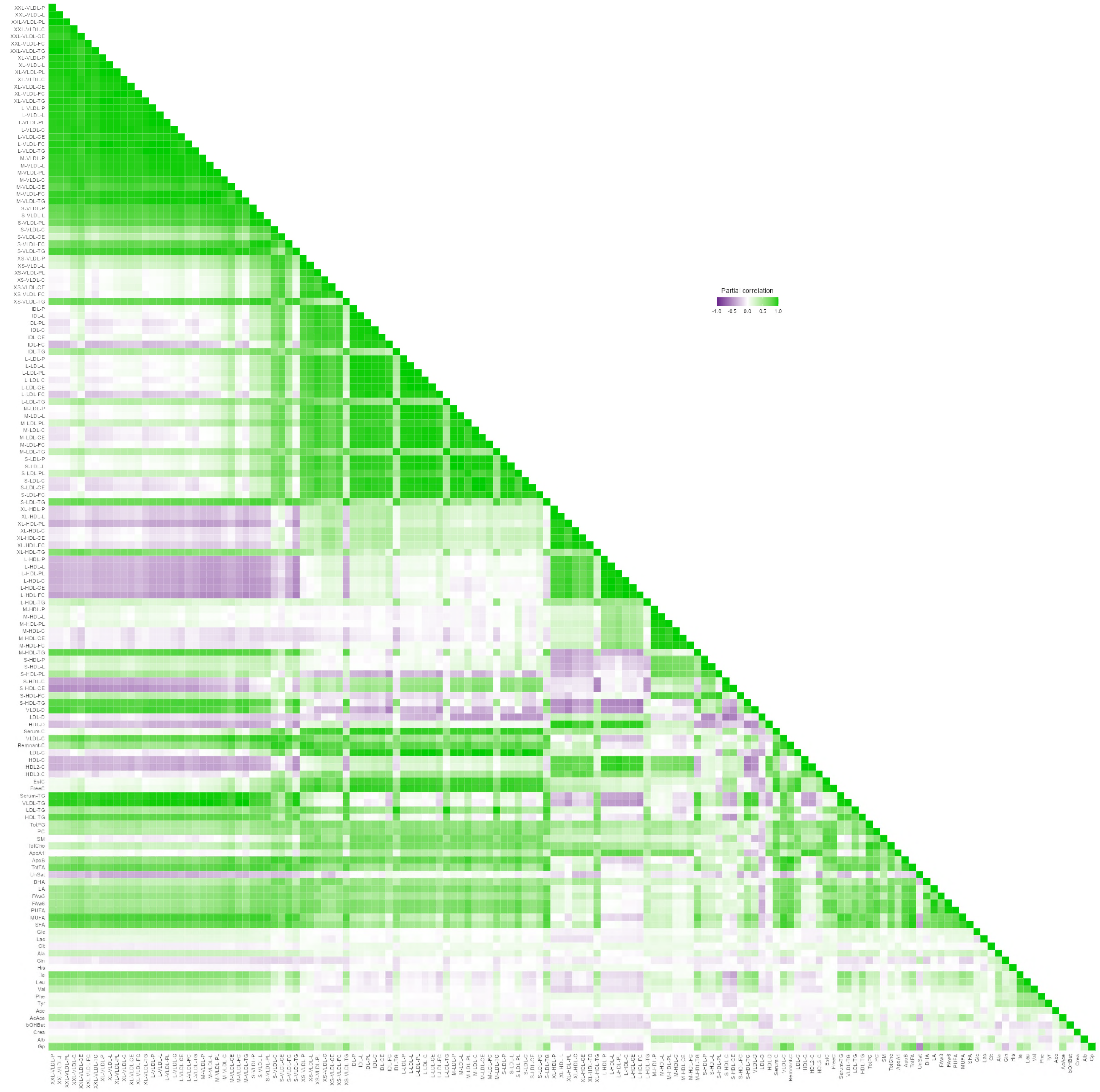

Supplementary Figure S2. Associations of directly-measured metabolic biomarkers with incident type 2 diabetes

Adjusted for age, sex, study area, education, fasting time

Dashed line: p=0.05; Solid line: Bonferroni corrected significance threshold

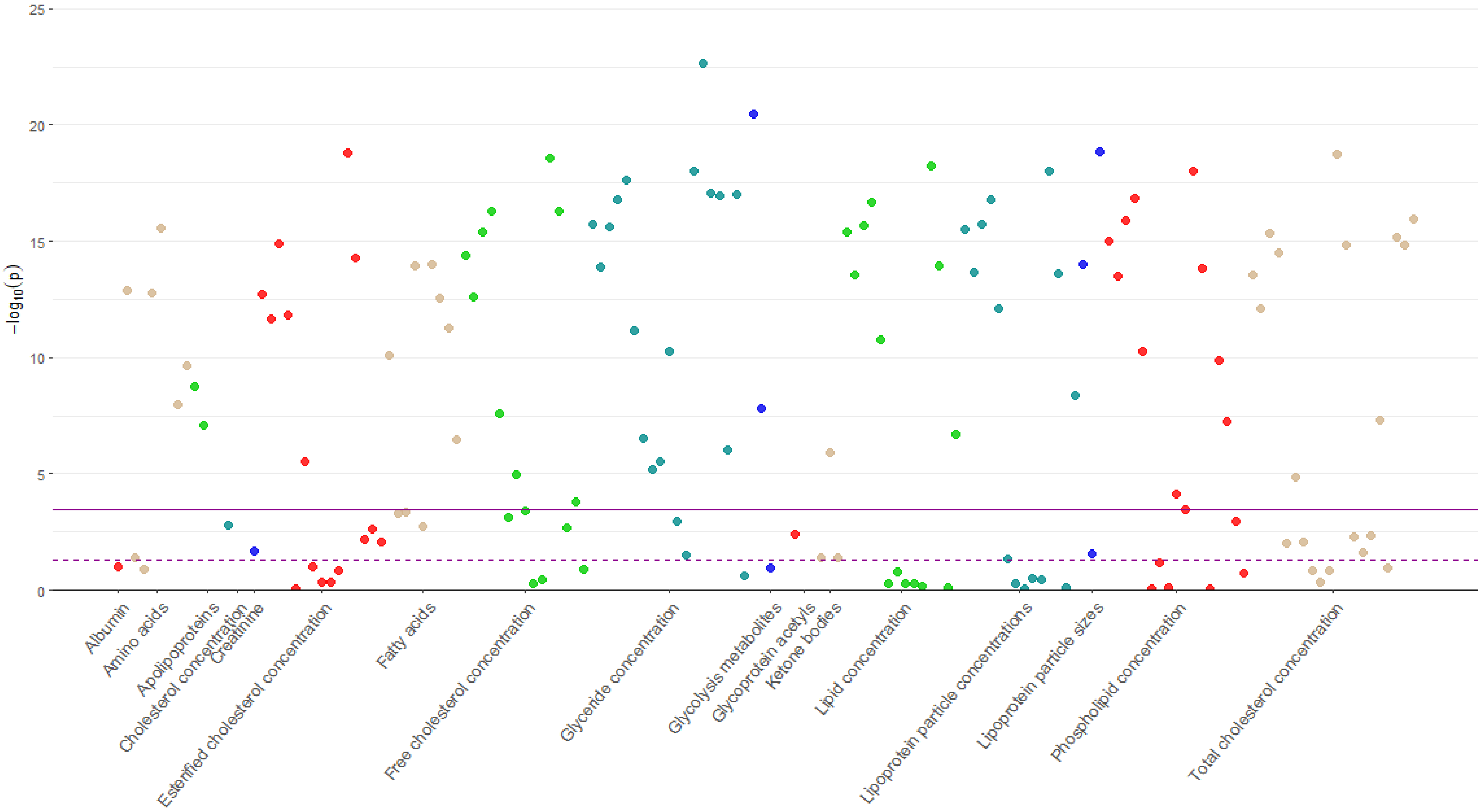

Supplementary Figure S3. Associations of metabolic biomarkers with incident type 2 diabetes

Adjusted for age, sex, region, education and fasting time

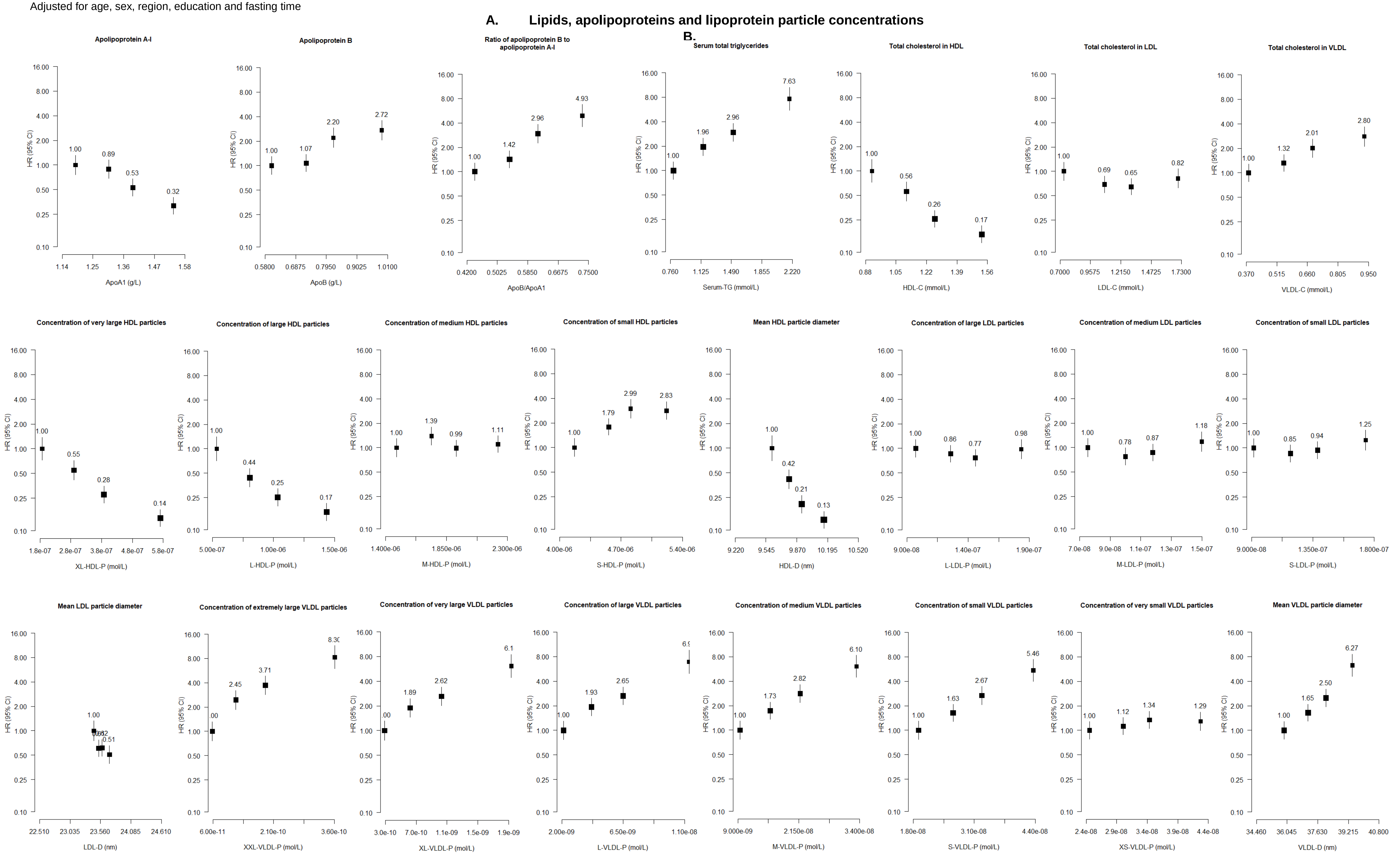

C. Fatty acids

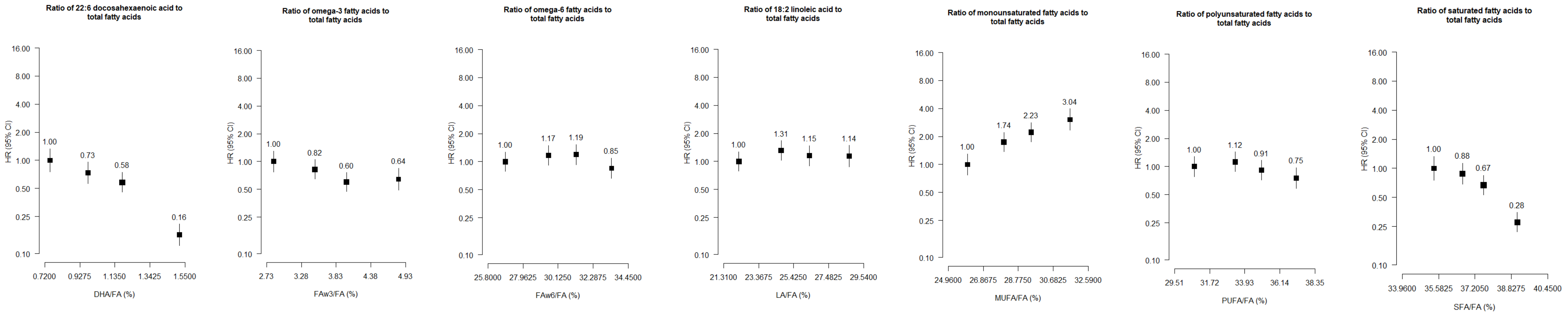

D. Amino acids

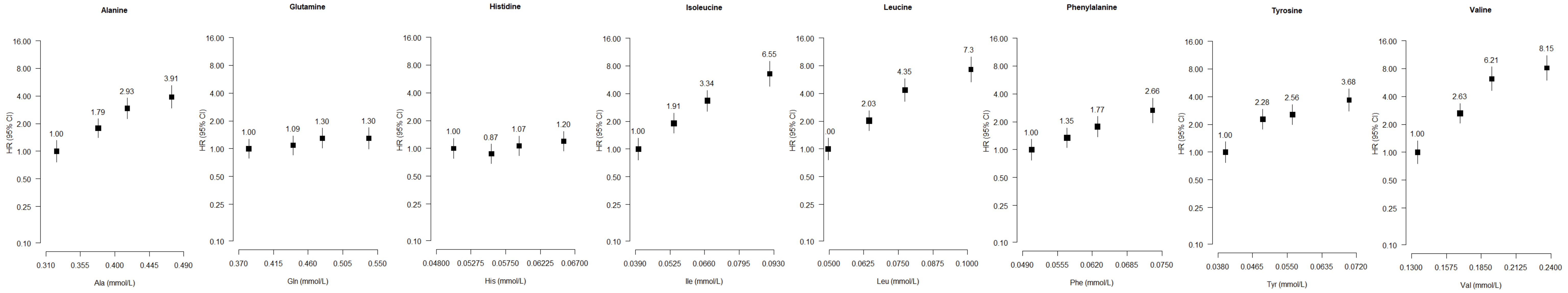

E. Glycolysis metabolites

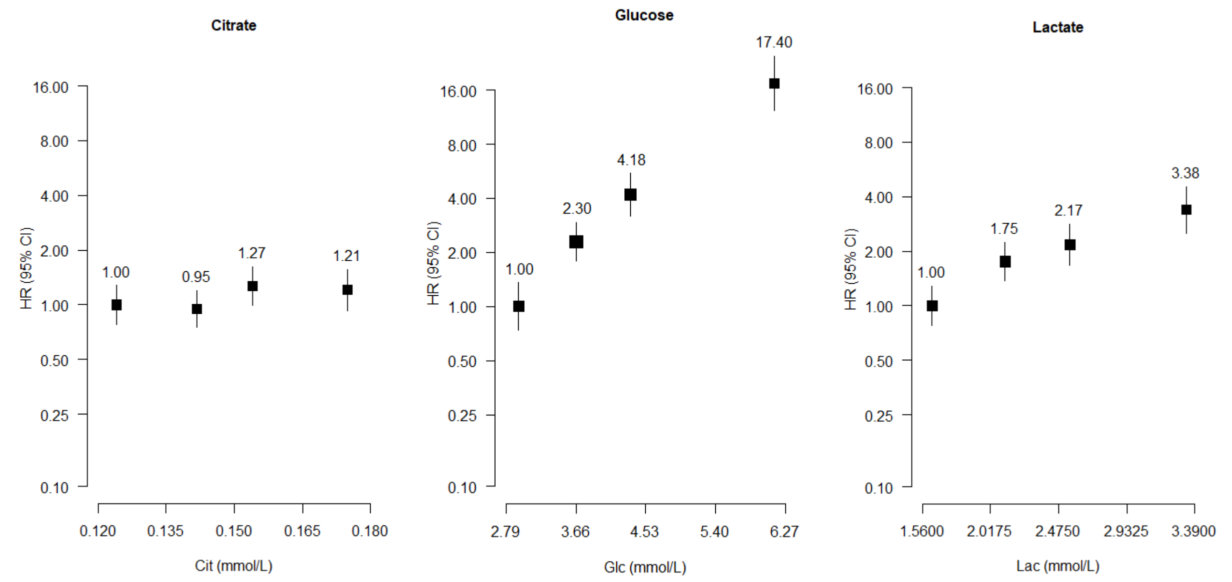

F. Ketone bodies

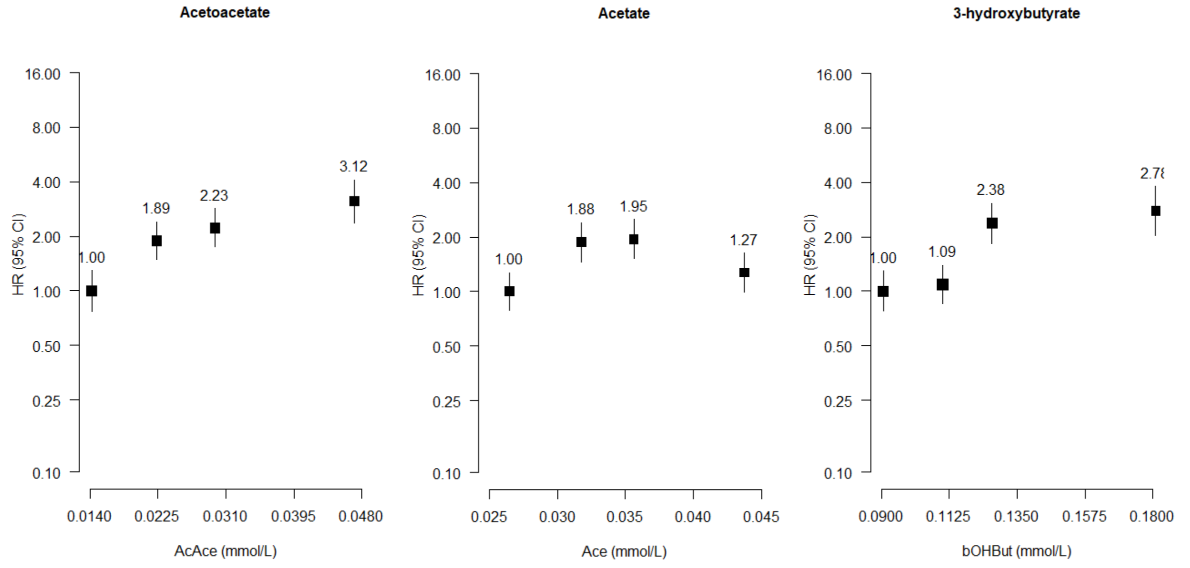

F. Inflammation

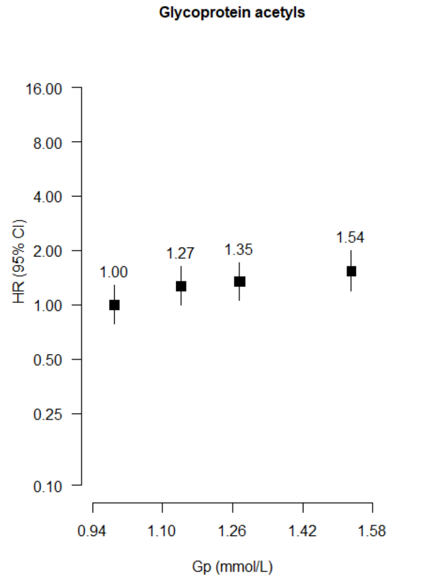

Supplementary Figure S4. Associations of lipids, apolipoproteins and lipoprotein particle concentrations with risk of incident type 2 diabetes, stratified by adiposity

Adjusted for age, study area, education, fasting time, smoking, alcohol, physical activity, dietary factors and family history of diabetes

p-values for heterogeneity (comparing estimates across BMI or waist circumference strata) after adjustment for multiple testing using Benjamini-Hochberg correction: *p≤0.05, **p≤0.01, ***p≤0.001

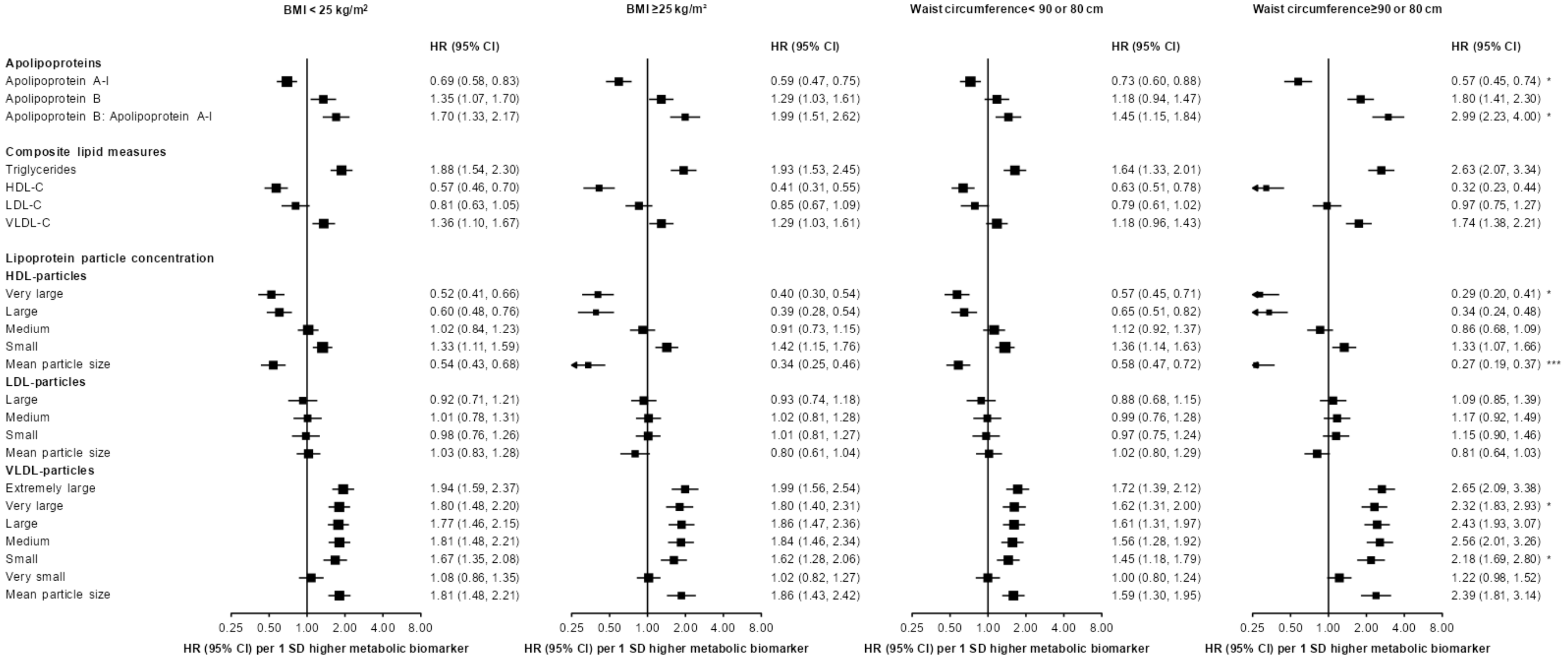

Supplementary Figure S5. Associations of fatty acids, amino acids, glycolysis metabolites, ketone bodies and inflammatory markers with risk of incident type 2 diabetes, stratified by adiposity

Adjusted for age, study area, education

p-values for heterogeneity (comparing estimates across BMI or waist circumference strata) after adjustment for multiple testing using Benjamini-Hochberg correction: *p≤0.05, **p≤0.01, ***p≤0.001

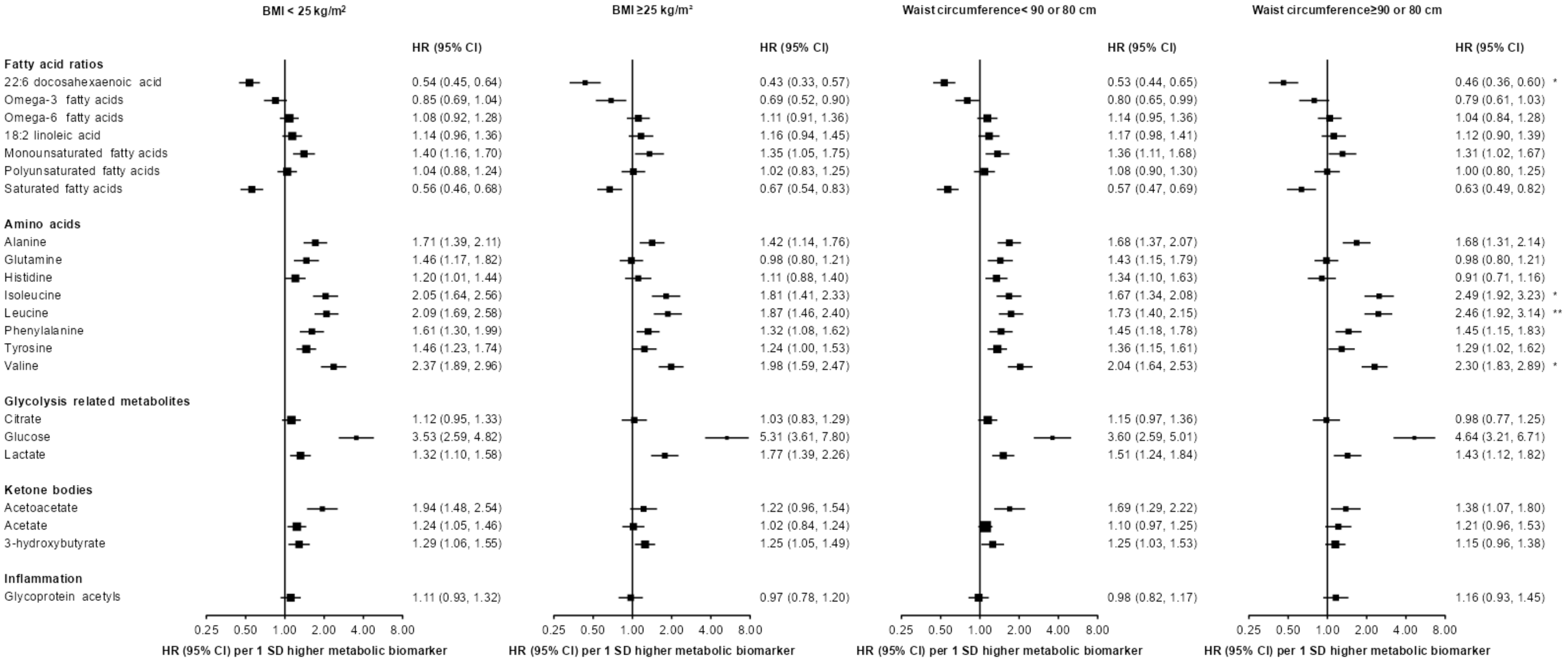

Supplementary Figure S6. Associations of lipids, apolipoproteins and lipoprotein particle concentrations with risk of incident type 2 diabetes, stratified by age

Adjusted for age, study area, education, fasting time, smoking, alcohol, physical activity, dietary factors, family history of diabetes, BMI and waist circumference

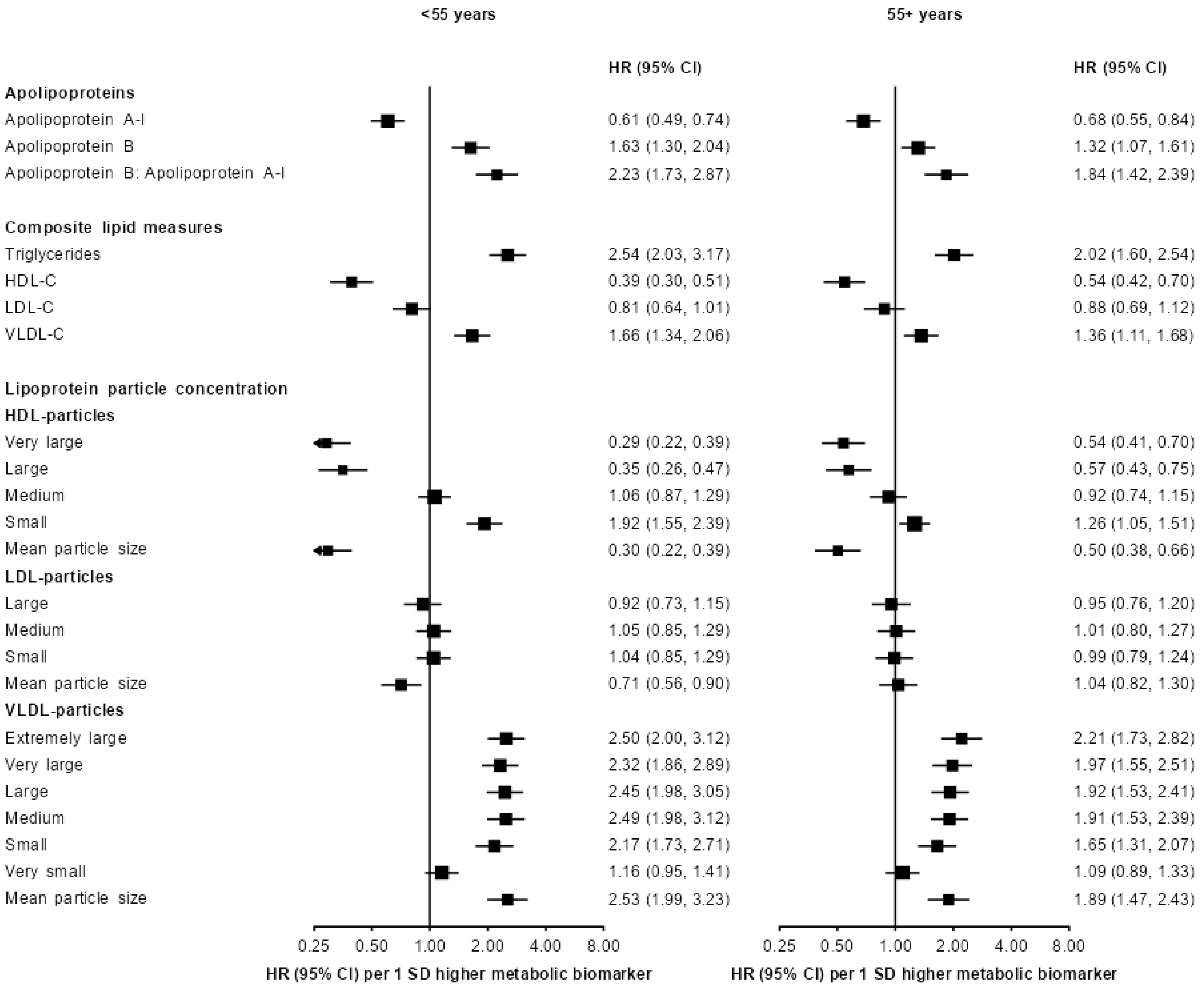

Supplementary Figure S7. Associations of fatty acids, amino acids, glycolysis metabolites, ketone bodies and inflammatory markers with risk of incident type 2 diabetes, stratified by age

Adjusted for age, study area, education, fasting time, smoking, alcohol, physical activity, dietary factors, family history of diabetes, BMI and waist circumference

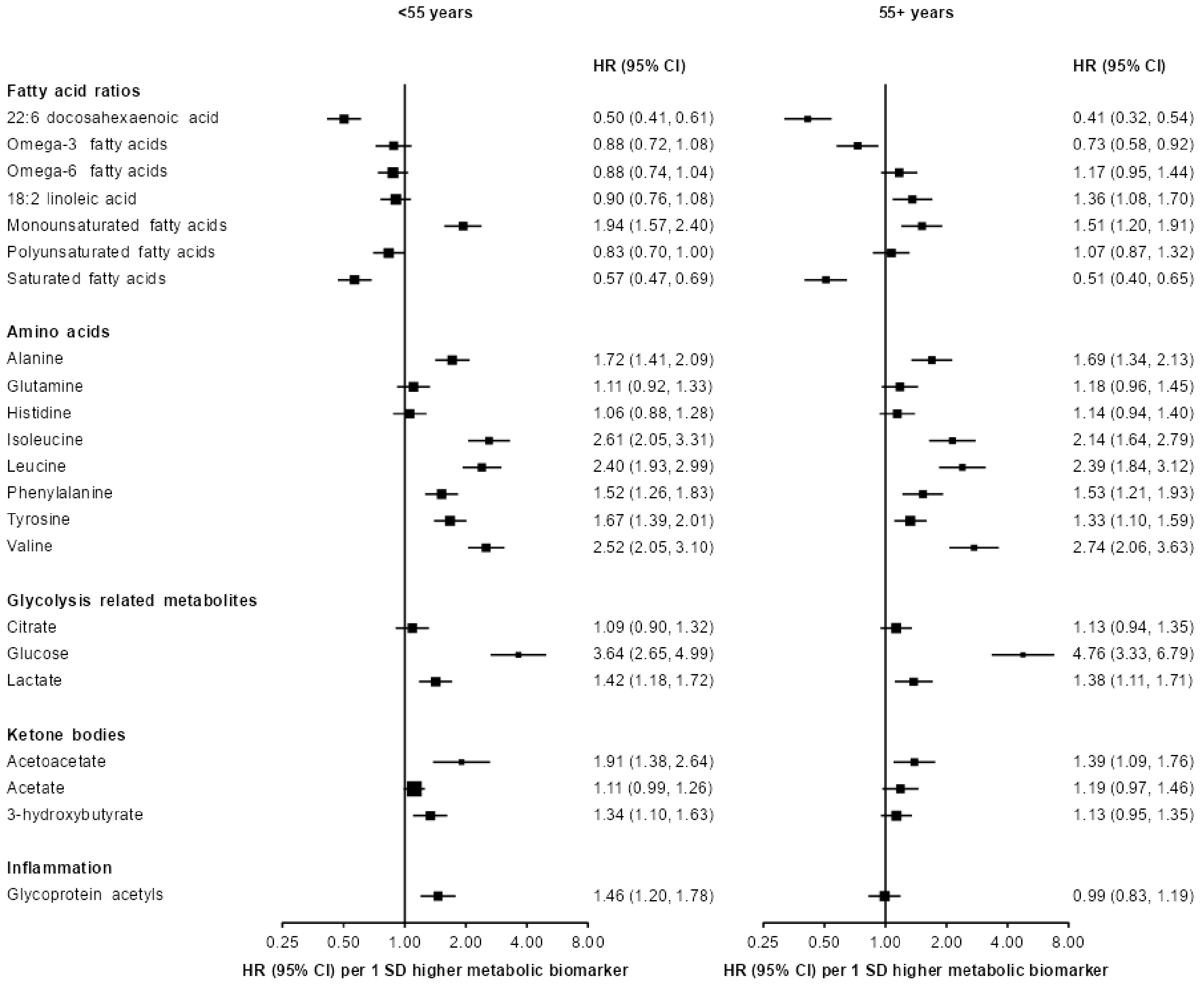

Supplementary Figure S8. Associations of lipids, apolipoproteins and lipoprotein particle concentrations with risk of incident type 2 diabetes, stratified by sex

Adjusted for age, study area, education, fasting time, smoking, alcohol, physical activity, dietary factors, family history of diabetes, BMI and waist circumference

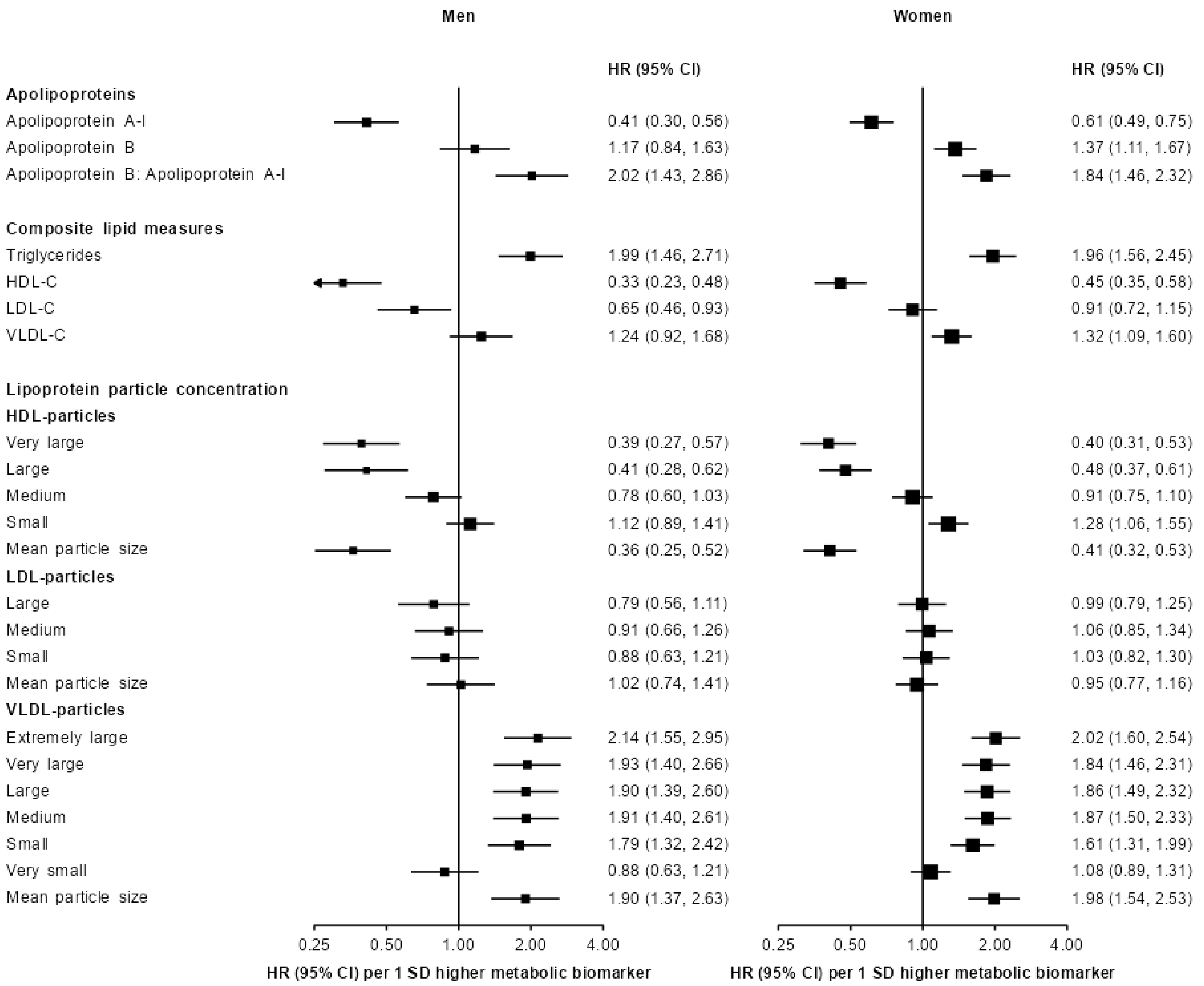

Supplementary Figure S9. Associations of fatty acids, amino acids, glycolysis metabolites, ketone bodies and inflammatory markers with risk of incident type 2 diabetes, stratified by sex

Adjusted for age, study area, education, fasting time, smoking, alcohol, physical activity, dietary factors, family history of diabetes, BMI and waist circumference

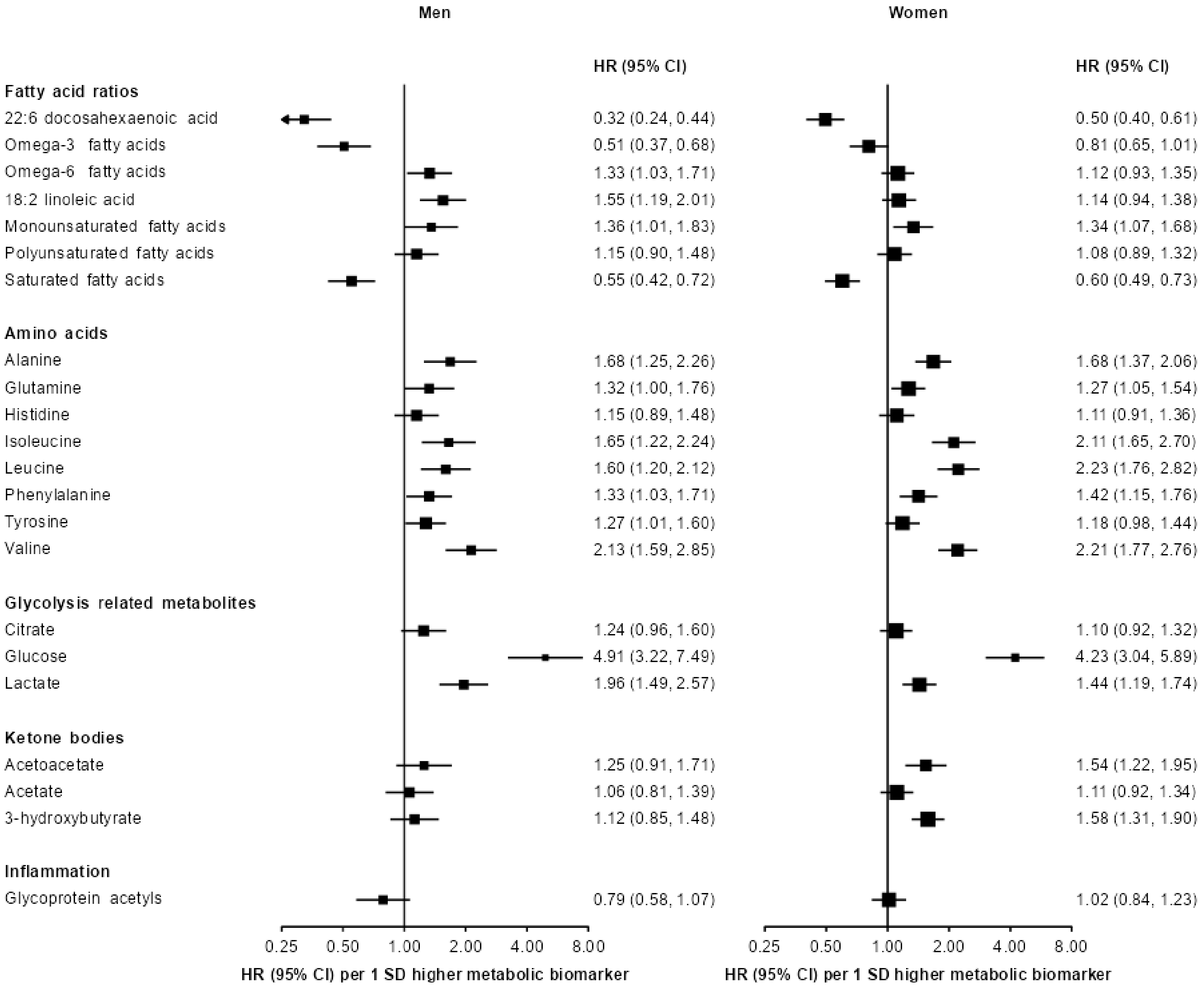

Supplementary Figure S10. Associations of lipids, apolipoproteins and lipoprotein particle concentrations with risk of incident type 2 diabetes, stratified by region

Adjusted for age, study area, education, fasting time, smoking, alcohol, physical activity, dietary factors, family history of diabetes, BMI and waist circumference

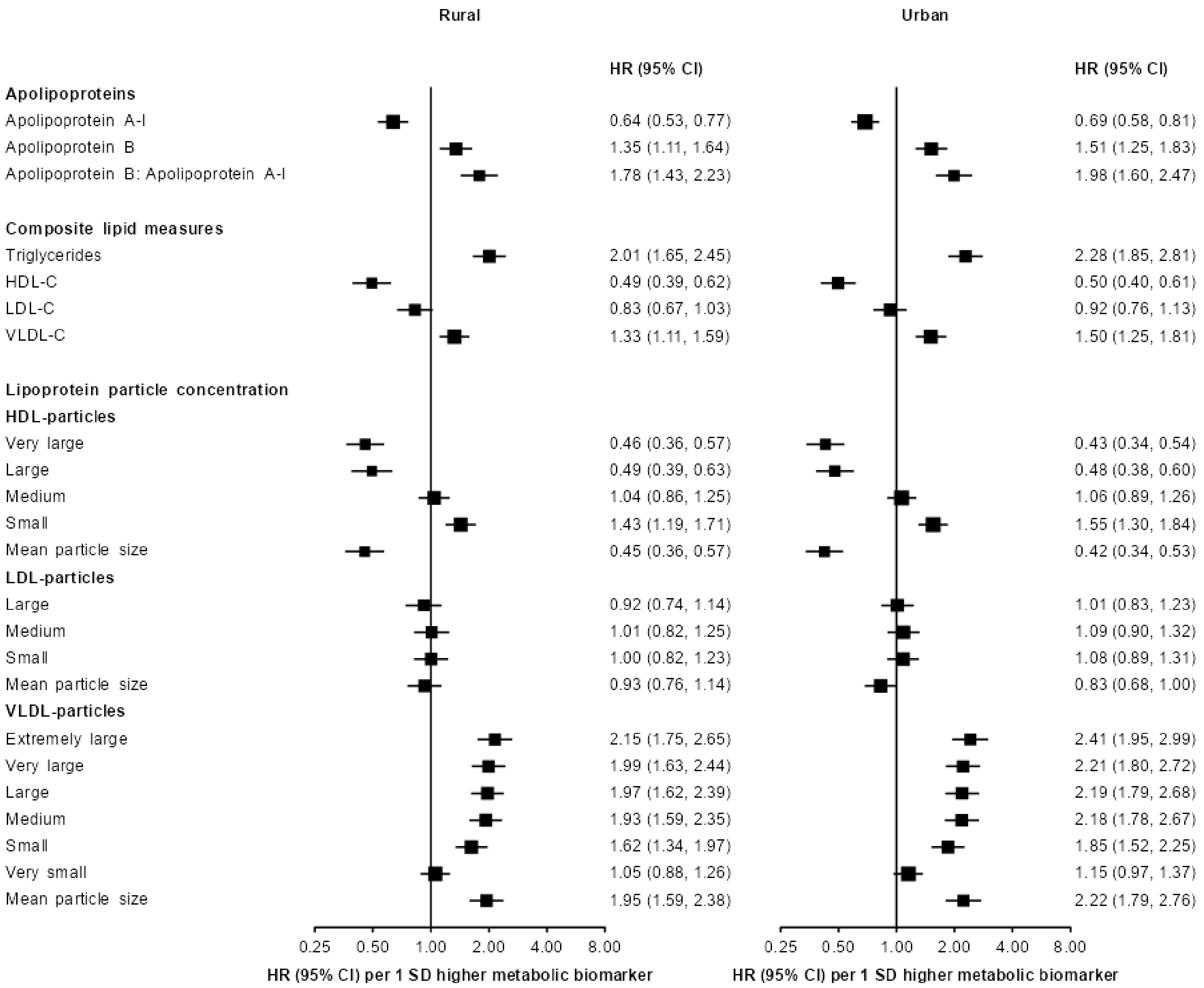

Supplementary Figure S11. Associations of fatty acids, amino acids, glycolysis metabolites, ketone bodies and inflammatory markers with risk of incident type 2 diabetes, stratified by region

Adjusted for age, study area, education, fasting time, smoking, alcohol, physical activity, dietary factors, family history of diabetes, BMI and waist circumference, fasting time, smoking, alcohol, physical activity, dietary factors and family history of diabetes

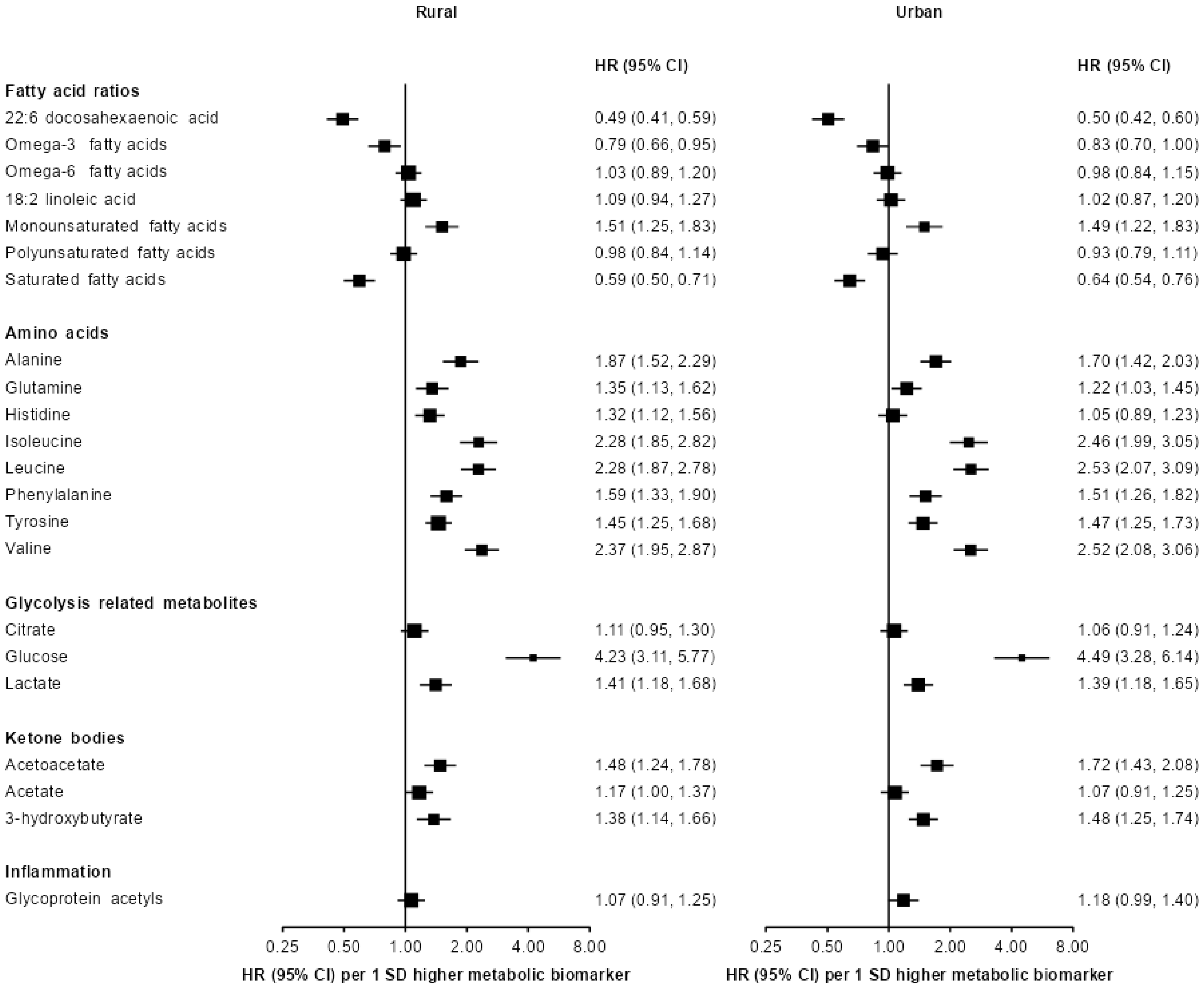

Supplementary Figure S12. Associations of metabolic biomarkers with risk of incident type 2 diabetes (n=757) excluding the first 2 years of follow-up

Adjusted for age, sex, study area, education, fasting time, smoking, alcohol, physical activity, dietary factors, family history of diabetes, BMI and waist circumference. Squares represent the HR. Horizontal lines represent the corresponding 95% CI. Fatty acid ratios represent ratios of individual to total fatty acids

*p≤0.05, **p≤0.01, ***p≤0.001 after adjustment for multiple testing using Benjamini-Hochberg correction

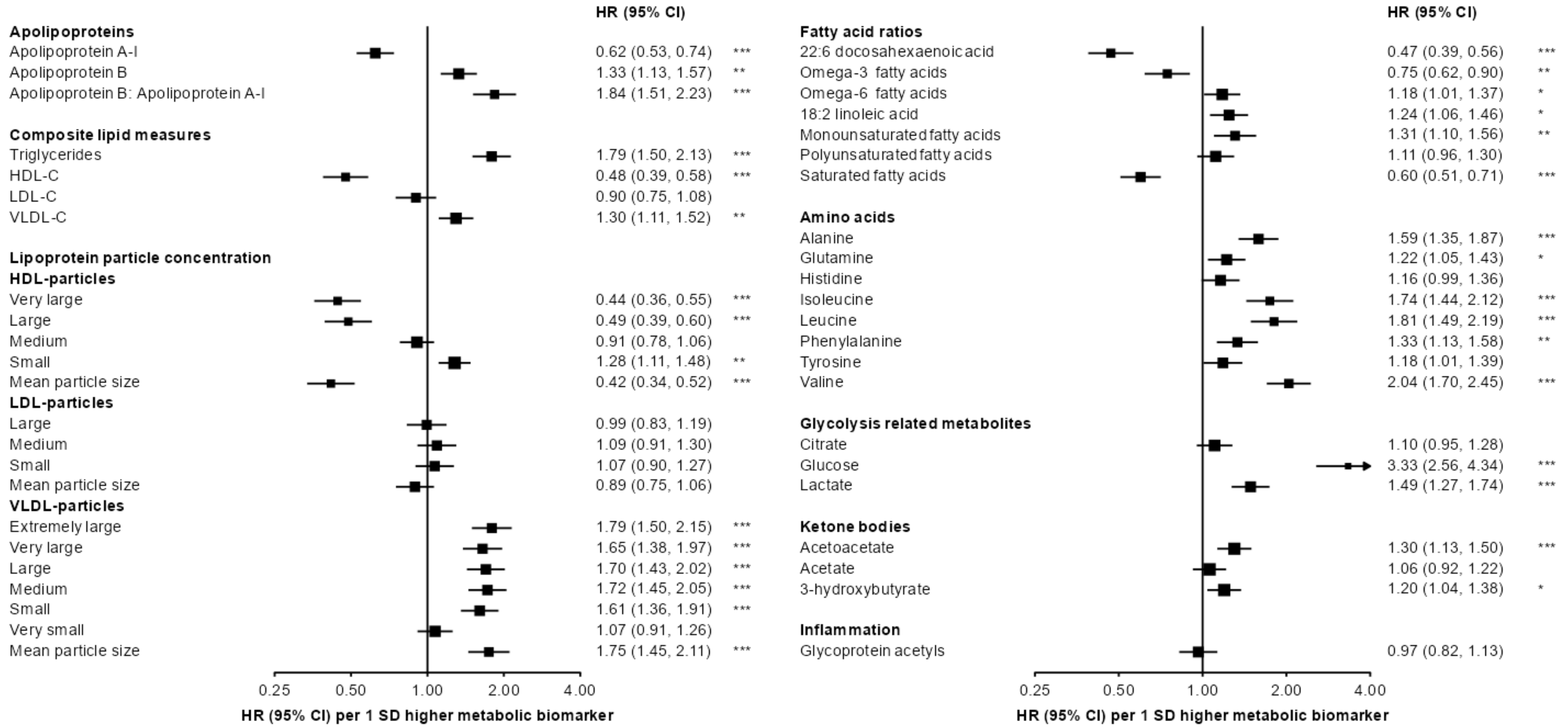

Supplement: Supplementary file 1 — Supplementary Information. [file 41598_2022_19159_MOESM1_ESM.pdf]
